# *DH2*-dependent trans-acting siRNAs regulate leaf and lemma development in rice

**DOI:** 10.3389/fpls.2024.1534038

**Published:** 2025-01-27

**Authors:** Jun Tang, Tianye Li, Yuanzhuo Gao, Xinghang Li, Ziheng Huang, Hui Zhuang, Guanghua He, Hongfa Luo, Yunfeng Li

**Affiliations:** ^1^ Key Laboratory of Application and Safety Control of Genetically Modified Crops, College of Agronomy and Biotechnology, Southwest University, Engineering Research Center of South Upland Agriculture, Ministry of Education, Chongqing, China; ^2^ College of Agronomy, Guizhou University, Guiyang, China

**Keywords:** AGO7, polarity establishment, ta-siRNAs, lateral organs, ARFs

## Abstract

In most crops, the development of lateral organs such as leaves and floral organs plays important roles in the architecture of plants and grains and then determines the yield. Establishment of polarity in these lateral organs is one of the most critical events for their morphogenesis. However, the molecular mechanisms regarding this in rice remain unclear. Here, we isolated two allelic mutants named *degenerated hull 2-1* and *degenerated hull 2-2* (*dh2-1* and *dh2-2*) in rice, exhibiting abaxially rolled leaves and rod-shaped lemmas. *DH2* encoded the relatively conservative ARGONAUT 7 (AGO7) protein in plants and expressed in the lateral organs including leaf and floral organs. In addition, the overexpression lines of *DH2* showed adaxially rolled leaves. Next, it was proved that *DH2* was involved in the synthesis of *tasiR-ARFs*, the expression level of which was decreased sharply in lateral organs of *dh2* mutants. Then, it was found that the expression of *OsARF2, OsARF3, OsARF14*, and *OsARF15*, the potential targets of *tasiR-ARFs*, was increased in lateral organs of *dh2* mutants. However, it was not expected that the results of *in situ* hybridization showed that the four *ARF* genes were not expressed in WT lemma, whereas they were all ectopically expressed in rod-shaped lemma in *dh2* mutants. Meanwhile, *tasiR-ARFs* were expressed in the whole lemma but not in the abaxial side. This means that there was no opposite expression of *tasiR-ARFs* and *ARFs* in the adaxial–abaxial of lemma. Therefore, according to our data, we believe that the pathway of OsAGO7–tasiR-ARFs in rice was more likely involved in the development of the whole lemma, not only the abaxial side, by restricting the ectopic expression of *OsARFs* in the whole lemma, which was different from that in the lateral organs of Arabidopsis.

## 1 Introduction

Seed plants have two different organ growth patterns: a root system and a shoot system (Bowman et al., 2002; Golz and Hudson, 2002). The shoot system includes stems and lateral organs (leaves and flowers), and lateral organs are formed by the lateral flanks of the shoot apical meristem (SAM) and then exhibit lateral growth and significant asymmetric development along three (proximal–distal, adaxial–abaxial, and medial–lateral) axes (Moon and Hake, 2011). Rice leaves are the main organ of photosynthesis, and improving leaf morphology is one of the effective ways to increase yield (Xu et al., 2018). The flower organs in rice eventually develop into grains, in which lemma and palea limit grain shape and size and then affect grain yield to a great degree (Xiang et al., 2015; Yu et al., 2018). Therefore, the development of leaves and floral organs has an important role in the vegetative and reproductive growth stages of rice.

In the initial stage of lateral organ formation, after founder cells that are designated to develop into lateral organs are recruited from the SAM, the three polarity axes—adaxial–abaxial, medial–lateral, and proximal–distal—are established in an orderly manner (Bowman et al., 2002; Braybrook and Kuhlemeier, 2010; Moon and Hake, 2011). The molecular mechanism underlying the establishment of lateral organs’ adaxial–abaxial polarity in Arabidopsis and rice has been extensively studied.

In Arabidopsis, three HD-ZIP III genes [*PHABULOSA (PHB)*, *PHAVOLUTA (PHV)*, and *REVOLUTE (REV)*] and a LOB-domain (LBD) transcription factor, *AS2*, all predominantly express on the adaxial side and regulate adaxial cell development in leaves. The expression of HD-ZIPIII genes was restricted by *miR165/166*, which accumulate on the abaxial side (Tang et al., 2003; Juarez et al., 2004). Then, dominant mutations of HD-ZIPIII genes cause a lateral organ adaxialization (McConnell et al., 2001; Prigge et al., 2005; Otsuga et al., 2008). In rice, adaxially rolled leaves were commonly observed in the plants expressing *OSHB1m*, *OSHB3m*, and *OSHB5m* (*microRNA165/166*-resistant HD-ZIPIII gene versions) genes, implying that the *miR165/166-*HD-ZIP III gene pathway is key to adaxial cell fate and conserved in plants (Itoh et al., 2008; Li et al., 2016; Zhang et al., 2021). However, although *OsAS2* overexpression resulted in defects of leaf structure, it is likely that the adaxial–abaxial patterning is not affected obviously. In addition, the *OsAS2* transcripts were identified throughout leaf primordia. Thus, either *OsAS2* in rice or *AS2* in Arabidopsis may have a divergent role in adaxial polarity establishment of leaf (Ma et al., 2009).

It has been reported that *KANADIs* (*KAN)*, *YABBYs* (*YAB*), and *ARFs* family genes are involved in the establishment of the abaxial polarity of lateral organs. In Arabidopsis, *KAN1*, *KAN2*, *YAB1*, *YAB2*, *YAB3*, and *ARF2*, *ARF3/ETT*, and *ARF4* are all expressed in the abaxial side, the mutations of which lead to adaxialization or reduced abaxial identity in lateral organs (Yu et al., 2017; Eshed et al., 2001; Kerstetter et al., 2001; Emery et al., 2003; Sawa et al., 1999; Siegfried et al., 1999; Stahle et al., 2009; Sarojam et al., 2010; Pekker et al., 2005; Guan et al., 2017). In rice, *KAN-like* gene *SHALLOT-LIKE1* (*SLL1*) accumulates in the abaxial epidermis of leaf and regulates leaf abaxial cell development (Zhang et al., 2009). *OsYAB1* is expressed in the abaxial sclerenchyma in the leaves, and the palea and lemma, and the mutation of *OsYAB1* causes the abaxial-rolling leaves. However, *OsYAB1* was recognized as a regulator of sclerenchymatous cell types but not as an abaxial polarity regulator (Toriba et al., 2007; Dai et al., 2007). A recently study found that *SLL1* could directly regulate the proper expression of *OsARF2/OsARF3a/OsETT* and then contribute to lemma polarity development in rice (Si et al., 2022).

It has been broadly reported that *TAS3 trans-acting small interfering RNAs* (*ta-siRNAs*, generally termed *tasiR-ARFs*) participate in specifying abaxial–adaxial fates, which express in the adaxial domain and represses the expression of *ARFs* there (Garcia et al., 2006; Takahashi et al., 2013; Adenot et al., 2006). During the generation of *tasiR-ARFs*, *TAS3* transcripts are firstly cleaved by miR390 and ARGONAUTE7 (AGO7) into fragments, which are then converted to double-stranded RNA by RNADEPENDENT RNA POLYMERASE 6 (RDR6) with SUPPRESSOR OF GENE SILENCING 3 (SGS3), and then are further processed into 21-nucleotide *tasiRNAs* by DICER-LIKE 4 (DCL4) (Liu et al., 2020; Song et al., 2019, 2012a, 2012b; Toriba et al., 2010; Yoshikawa et al., 2005; Liu et al., 2007; Si et al., 2022). Finally, mature *tasiR-ARFs* are loaded into AGO1 protein to target ARFs (Allen et al., 2005; Xie et al., 2004; Yoshikawa et al., 2005; Si et al., 2022). Therefore, these genes related to the *tasiR-ARFs* generation are also involved in the establishment of abaxial–adaxial polarity. In rice, the mutations of *AGO7*, *OsSGS3a*/*PDL1, SHOOTLESS2(SHL2)/OsRDR6*, and *SHO1/OsDCL4* exhibit obvious defects in polarity development of lateral organs, especially in lemma (Zhang et al., 2023; Itoh et al., 2000; Nagasaki et al., 2007; Liu et al., 2007; Toriba et al., 2010; Song et al., 2012). These mutants all display reduced accumulation of *tasiR-ARFs* and overexpression/ectopic expression of *ARF* genes. However, because of too many genes in this pathway, the molecular mechanism of *AGO7–tasiR-ARFs–ARFs* in regulating the polarity development of lateral organs in rice still remains unresolved.

In this study, we characterized two polarity defects of lateral organ mutants named *degenerated hull 2-1* and *degenerated hull 2-2* (*dh2-1* and *dh2-2*), which exhibit abaxial leaves and rod-like lemmas caused by defects in polarity development. *DH2* encodes the AGO7 protein and is highly expressed in the lateral organs including leaf and floral organs. Our data indicate that *DH2* is involved in *tasiR-ARFs* synthesis, thereby affecting the organ but not polarity distribution of *ARFs* transcript in lateral organs, like lemma in rice. Therefore, our findings provide new views about the mechanism level that shows how *DH2*-mediated *tasiR-ARFs* pathways regulate lateral organ development in rice.

## 2 Materials and methods

### 2.1 Plant materials

The *dh2-1* and *dh2-2* mutants were respectively isolated from an ethylmethane sulfonate-treated population of Xian cultivar “Jinhui 10” and “Xi Da 1B”, which was used as the wild type (WT). The *dh2-1* mutant was used for rice genetic transformation, among which transgenic plants exhibit overexpression of *DH2*. The plant materials used in other experiments were grown in experimental fields of Southwest University in Chongqing, China.

### 2.2 Morphological and histological analysis

Leaf at the seedling stage, panicles at the heading stage, and spikelets at the flowering stage of *dh2-1*, *dh2-2*, and WT (J10, 1B) were used to investigate the phenotypic characteristics. Fresh material from the field was taken, stuck on the conductive adhesive, and observed. A Nikon SMZ1500 stereomicroscope (Nikon, Tokyo, Japan) and a Hitachi SU3500 scanning electron microscope (Hitachi, Tokyo, Japan) were used (low vacuum, −20°C, 5 kV). The leaves of WT (J10) and *OE-DH2* lines, *dh2-1*, and *dh2-2* at the seedling stage were observed using a Nikon SMZ1500 stereomicroscope (Nikon, Tokyo, Japan).

For paraffin sectioning, tissues of the *dh2-1*, *dh2-2*, WT (J10, 1B), and *OE-DH2* lines were collected in FAA solution (total 50 mL volume: 5 mL of 37% formaldehyde, 2.5 mL of 0.9 M glacial acetic acid, 25 mL of ethanol, and 17.5 mL of ddH_2_O) for a suitable time (24–48 h) after vacuum infiltration in room temperature. The fixed rice tissues were dehydrated in an ethanol gradient series (50% ethanol, 70% ethanol, 85% ethanol, 95% ethanol, and three times 100% ethanol), for approximately 30–60 min each time. After alcohol treatment, the materials were treated with a 3:1, 1:1, and 1:3 ratio of alcohol and xylene, as well as three times of 100% xylene, for 30–60 min each time. In order to embed the materials in paraffin, the materials were mixed with xylene and melted paraffin (1:1) (Sigma, St. Louis, MO, USA) in a 42°C oven overnight, as well as four rounds of paraffin in as 65°C oven for 6–12 h each time. Samples were sectioned into 10-μm thickness and stained with 1% safranin and 1% Fast Green (Amresco), and observed using an Eclipse E600 light microscope (Nikon).

### 2.3 Phenotypic statistics

The leaf rolling index (LRI) and leaf erect index (LEI) of the top three leaves (flag leaf, second upper leaf, and third upper leaf) were determined at the grain-filling stage as described previously (Shi et al., 2007; Zhang et al., 2009). All the phenotypic data shown in this study were measured using 1B, *dh2-1, dh2-2*, J10, and two or three independent transgenic lines (*DH2-OE*), and 10 individual plants per line were used for the measurement.

### 2.4 Anatomical observation of leaves

Take leaves of 30-day-old seedlings of 1B, *dh2-1, dh2-2*, J10, and two or three independent transgenic lines (*DH2-OE*) from the field, embed them with a frozen embedding agent, and make slices of approximately 10 µm thickness on a Leica frozen slicer; the leaf slices were directly imaged using visible or ultraviolet light excitation, under an Eclipse E600 light microscope (Nikon).

### 2.5 Genetic and complementary analysis of *DH2*

Gene message information was downloaded from the Gramene (http://www.gramene.org/). The BLAST tool on the National Center for Biotechnology Information website was used to conduct homology analysis. To construct the complementary vector of *DH2*, a fragment containing a 7,623-bp WT genomic fragment of *DH2* was amplified using the primers *DH2*-com-F1, *DH2*-com-F2, *DH2*-com-R1, and *DH2*-com-R2 (a 7,623-bp WT genomic fragment was divided into two segments and amplified separately). DNA extracted from J10 was used to prepare PCR amplification template using Prime STAR^®^ Max DNA Polymerase (TaKaRa, Dalian, China). The two PCR amplification fragments (F1/R2 to amplify fragment 1 and F2/R1 to amplify fragment 2) were purified by a universal DNA Purification Kit (TianGen, Beijing, China) and recombined into pCAMBIA1300 using the Minerva Super Fusion Cloning Kit (UElandy, Suzhou, China). After conducting sequence analysis for verification purposes, the vector was subjected to *dh2-1* using the *Agrobacterium tumefaciens*-mediated technique. The primer sequences used are listed in **Supplementary Table S1**. All the experiments above were performed according to the manufacturer’s protocol.

### 2.6 RNA isolation and RT-qPCR analysis

Total RNA was extracted from individual organs using the KK Fast Plant Total RNA Kit (Zoman, Beijing, China) and was used to synthesize the first-strand cDNA by using the UEIris RT mix with DNase (All-in-One) (UElandy, Suzhou, China). RT-qPCR analysis was performed using the SYBR^®^ Premix ExTaq™ II 4 Kit (TaKaRa, Dalian, China) in a CFX ConnectTM Real-Time System (BIO-RAD, USA). To obtain the average expression levels of each gene, at least three replicates were examined. *ACTIN* (*LOC_Os03g50885*) and *U6* were used as endogenous controls.

### 2.7 *In situ* hybridization

*In situ* hybridization was performed according to previous methods (Zhang et al., 2017). The DIG RNA Labeling Kit (Roche, Basel, Switzerland) was used to label the gene-specific *OsARF2*, *OsARF3, OsARF4, OsARF14*, and *OsARF15* probe. A *TAS* complementary LNA-modified DNA was used as the *TAS* detection probe. Shanghai Jie-Li Biotechnology Company synthesized a *TAS* complementary LNA-modified DNA probe. The primer sequences used are listed in **Supplementary Table S1**.

### 2.8 Protein sequence and phylogenetic analysis

GenBank (http://www.ncbi.nlm.nih.gov/genbank/) was used to download the 12 AGO7 protein sequences from different plant species by blasting *DH2* candidate gene *Os03g33650* as a query. GenBank (http://www.ncbi.nlm.nih.gov/genbank/) was used to download the 21 AGO protein sequences between rice and Arabidopsis by blasting *DH2* candidate gene *Os03g33650* as a query. GenBank (http://www.ncbi.nlm.nih.gov/genbank/) was used to download 19 ARF protein sequences between rice and Arabidopsis by blasting these ARF genes as a query. The MEGA 10.0 software was used to construct the phylogenetic tree by the neighbor-joining method with 1,000 replicates (Tamura et al., 2011). MEME (http://alternate.meme-suite.org/) was used to predict the protein motifs. Accession numbers and plant species are listed in **Supplementary Table S2**.

## 3 Results

Two allelic recessive mutants of *DH2*, designated *dh2-1* and *dh2-2*, were identified in this study. Given that *dh2-1* showed typical defects, the remainder of this study focuses on the *dh2-1* mutants.

### 3.1 *dh2* mutants exhibited defective lemmas

**Figure 1:**
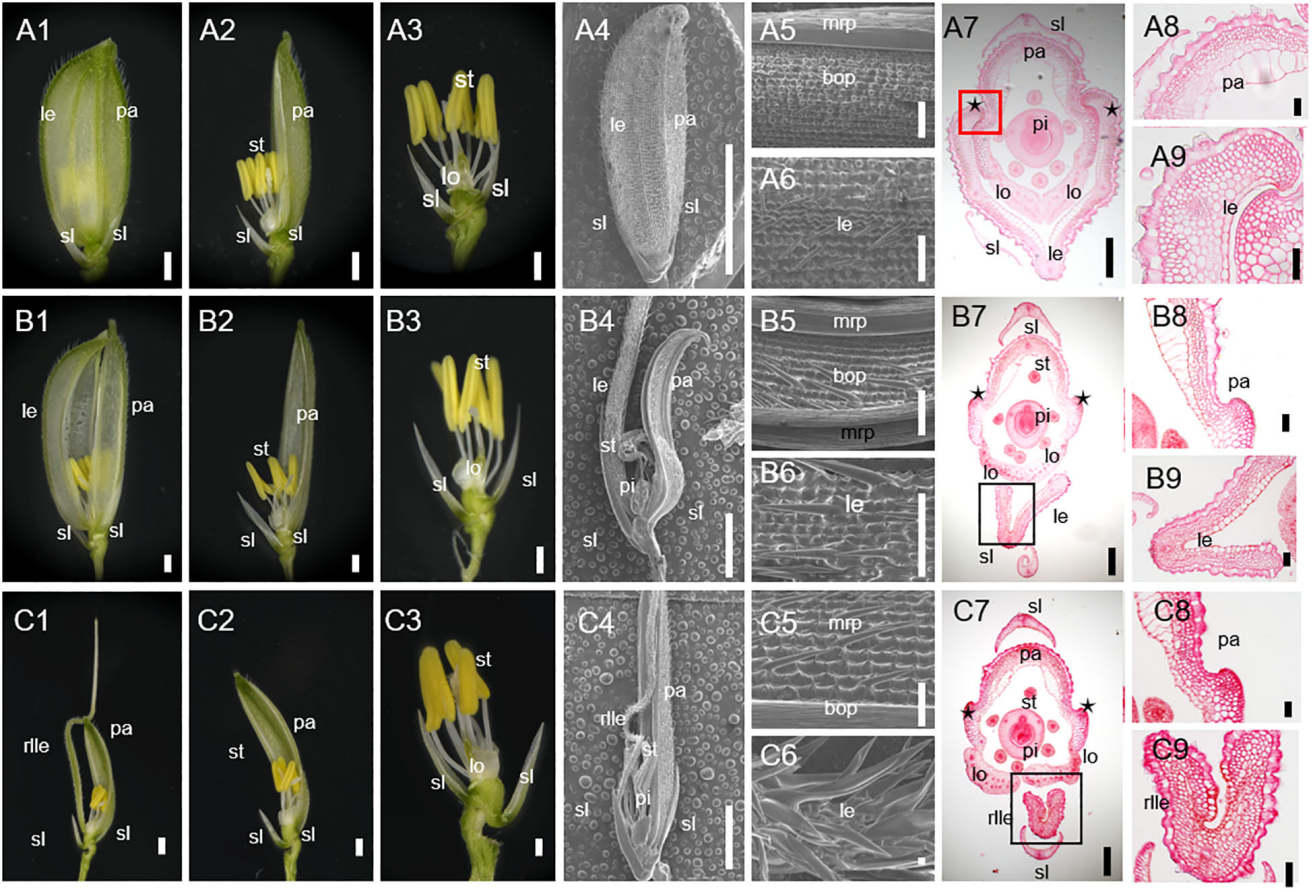
Phenotypes of spikelet in the wild-type (WT) and *degenerated hull 2-1* (*dh2-1*). **(A1)** The complete spikelet of the WT. **(A2)** The lemma was removed. The lemma and palea were removed in A3. **(A4)** The complete spikelet of the WT. **(A5, A6)** Surface characteristics of palea **(A5)** and lemma **(A6)** of WT spikelet. **(A7)** Cross-section of WT. **(A8, A9)** Transverse sections of palea **(A8)** and lemma **(A9)** of WT spikelet. **(B1)** The complete spikelet of the *dh2-1* with a type I lemma. **(B2)** The lemma was removed. The lemma and palea were removed in B3. **(B4)** The complete spikelet of the *dh2-1* with a type I lemma. **(B5, B6)** Surface characteristics of palea **(B5)** and lemma **(B6)** of the *dh2-1* with a type I lemma. **(B7)** Cross-section of *dh2-1* with a type I lemma. **(B8, B9)** Transverse sections of palea **(B8)** and lemma **(B9)** of *dh2-1* with a type I lemma. **(C1)** The complete spikelet of the *dh2-1* with a type II lemma. **(C2)** The lemma was removed. The lemma and palea were removed in C3. **(C4)** The complete spikelet of the *dh2-1* with a type II lemma. **(C5, C6)** Surface characteristics of palea **(C5)** and lemma **(C6)** of the *dh2-1* with a type II lemma. **(C7)** Cross-section of *dh2-1* with a type II lemma. **(C8, C9)** Transverse sections of palea **(C8)** and lemma **(C9)** of *dh2-1* with a type II lemma. le, lemma; rlle, rod-like lemma; pa, palea; sl, sterile lemma; lo, lodicule; st, stamen; pi, pistil; mrp, marginal region of palea; bop, body of palea. Bars = 1,000 μm in **(A1–A3, A5, A6, B1–B3, B5, B6, C1–C3, C5, C6)**. Bars = 5 mm in **(A4, B4, C4)**. Bars = 200 μm in **(A7, B7, C7)**. Bars = 50 μm in **(A8, A9, B8, B9, C8, C9)**.

In rice, the WT spikelet was composed of a pair of rudimentary glumes, a pair of sterile lemmas, and a fertile terminal floret, which contained four whorls of floral organs from outside to inside. Lemma and palea were shell-shaped and have five and three vascular bundles, respectively. The lemma had two inward hook-like edges, whereas the palea had two inward hook-like structures between the body of palea and margin region of palea. Then, they hooked together perfectly to provide a protective environment for the internal floral organs (**Figures 1A1–A6**).

In all the *dh2-1* spikelets, it was observed that the lemma and palea could not be hooked together mainly because of defective lemma. Based on the lemma phenotype, then *dh2-1* spikelets were classified into two types. In type I spikelets, although it still showed a shell shape, the lemma became significantly narrower than that of the WT (**Figures 1B1–B6**). In type II spikelets, the whole lemma almost degraded into a rod-shaped awn structure, losing its shell-shaped structural characteristics and exposing the inner floral organs (**Figures 1C1–C6**).

For further clarifying the defects in *dh2-1* lemma, the histology was analyzed between WT and *dh2-1* mutants. Firstly, the lemma of WT had five vascular bundles and wider than the palea. However, the *dh2-1* lemma became narrower, because the numbers of cells and vascular bundles were significantly reduced (**Figures 1A8, A9**). Secondly, the WT lemma had different epidermis cell types between the abaxial and the adaxial sides. The abaxial epidermis covered a silicified cell layer bearing trichomes and protrusions, whereas the adaxial epidermis consists of a layer of vacuolated cells (**Figures 1A7–A9**). In lemma of type I spikelets, the abaxial and adaxial sides were similar to the WT. However, in lemma of type II spikelets, the ectopic silicified cells were observed in the adaxial epidermis, even some lemmas completely lost adaxial epidermis and showed rod-shaped with a silicified abaxial epidermis (**Figures 1B7–B9, C7–C9**). In general, the *dh2-1* mutant showed defective medial–lateral and adaxial–abaxial polarities of lemma to different degrees, which suggests that *DH2* plays a vital role in establishing lemma polarity.

### 3.2 The defects of lemma in *dh2-1* occurred in the early stage of spikelet development

**Figure 2:**
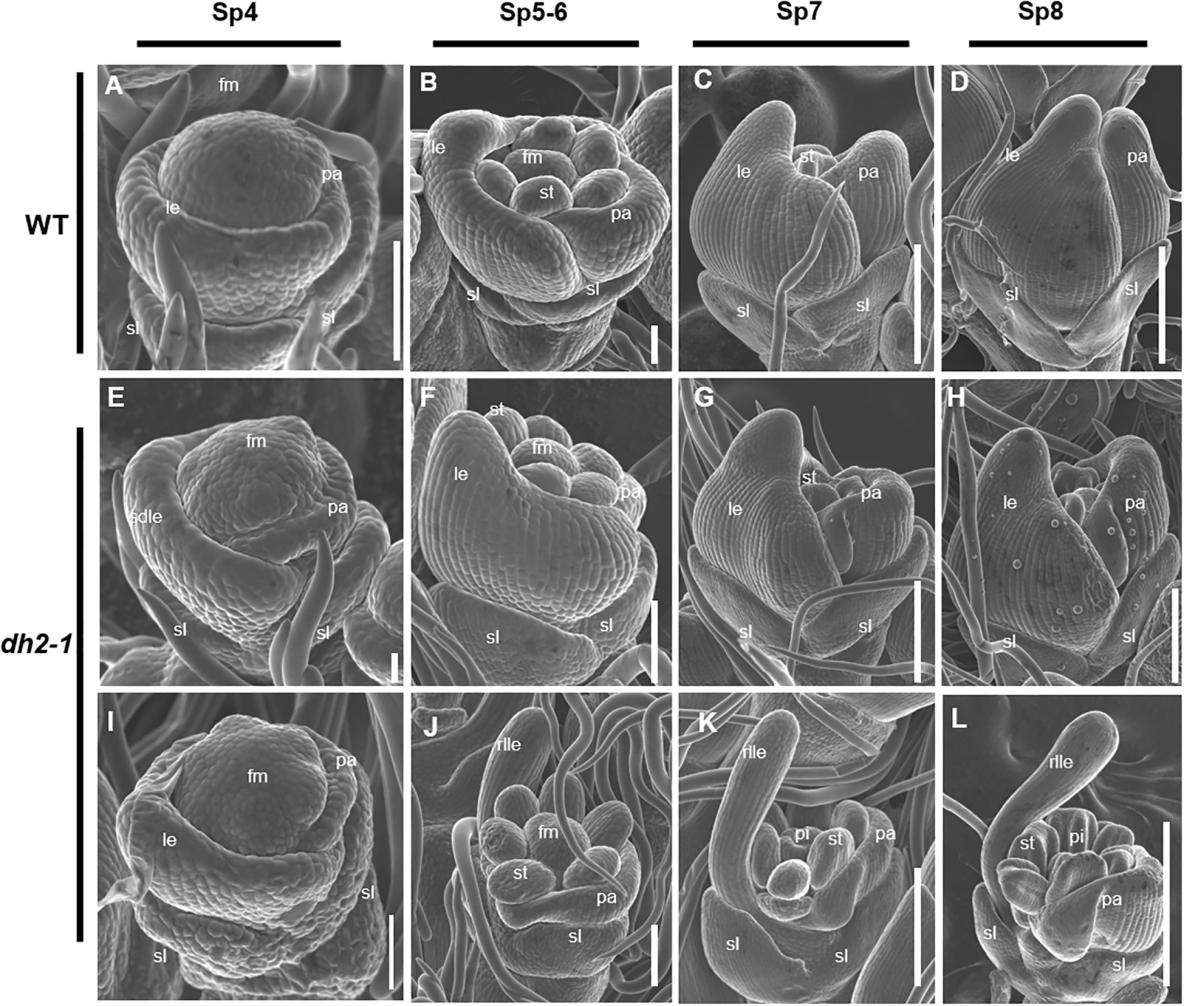
Scanning electron microscope images of florets at early developmental stages in the wild-type (WT) and *degenerated hull 2-1* (*dh2-1*). **(A–D)** WT. **(E, F)** Spikelet of *dh2-1* with a type I lemma. **(I–L)** Spikelet of *dh2-1* with a type II lemma. **(A, E, I)** Sp4; **(B, F, J)** Sp5–Sp6; **(C, G, K)** Sp7; **(D, H, L)** Sp8. sl, sterile lemma; le, lemma; rlle, rod-like lemma; pa, palea; st, stamen; pi, pistil; fm, flower meristem. Bars = 500 μm in A–L.

The development of WT and *dh2-1* mutants during the early spikelet development stage was observed by SEM. In the Sp4 stage, the lemma primordia had already formed and the palea primordia appears. In the Sp5–6 stage, while the lodicules and stamens primordia of the WT spikelet began to form, the lemma and palea primordia continued to differentiate, and the bottom edges of the palea and lemma began to hook together. Then, the lemma and palea primordia continued to differentiate and finally closed completely at the Sp8 stage (Ikeda et al., 2004). It should be pointed out that the lemma and palea were shell-shaped from the beginning of their primordia formation (**Figures 2A–D**).

At the Sp4 stage when lemma just formed, compared with WT, there were no obvious abnormalities in the *dh2-1* mutants (**Figures 2E, I**). However, from the Sp5 stage, the *dh2-1* spikelet displayed obvious defects. In the type I spikelet, the lemma began to become narrower in the medial–lateral direction than that of WT at the Sp5 stage, although it still showed a shell-shaped structure (**Figure 2F**). Next, from Sp6 to Sp8, it was observed that the lemma primordia could not differentiate enough in the medial–lateral direction and showed narrower than that of WT, resulting in the inner organ primordia being partially exposed (**Figures 2G, H**). In the type II spikelet, the lemma almost failed to differentiate in the medial–lateral direction at Sp5 and showed a rod-like form in its upper part (**Figure 2J**). Then, the lemma primordia of the *dh2-1* mutants further developed into a rod-like structure, and the stamens and pistil primordia were completely exposed from Sp7 to Sp8 (**Figures 2K, L**). Therefore, the entire data above indicated that the establishment of the medial–lateral and adaxial–abaxial polarities of lemma were seriously affected at the early stage of spikelet development, which was consistent with the observations at the mature stage.

### 3.3 *dh2-1* mutants exhibited defective leaves

**Figure 3:**
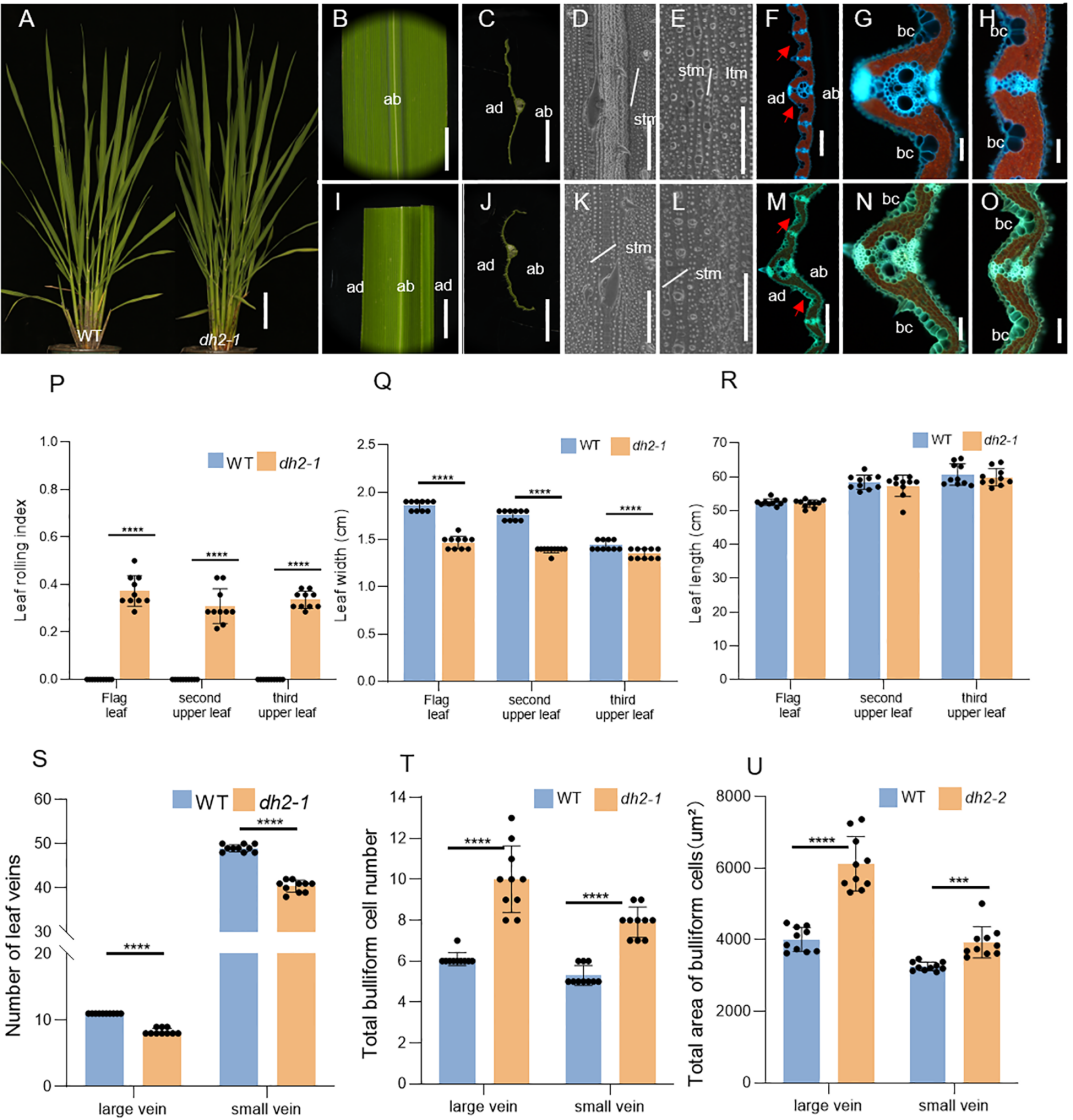
Phenotypes of leaves of WT and *dh2-1* mutant. **(A)** Phenotypes of the WT and *dh2-1* at the jointing stage. **(B)** Flag leaf of WT. **(C)** Free-hand cross-sections of WT flag leaf. **(D, E)** SEM observations of the adaxial **(D)** and abaxial **(E)** surface of leaves in WT. **(F)** Transverse sections of the leaf blade of WT at the seedling stage. **(G, H)** The high-magnification image of the area indicated by the red arrow in **(F, I)** Flag leaf of *dh2-1*. **(J)** Free-hand cross-sections of *dh2-1* flag leaf. **(K, L)** SEM observations of adaxial **(K)** and abaxial **(L)** surface of leaves in *dh2-1*. **(M)** Transverse sections of the leaf blade of *dh2-1* at the seedling stage. **(N, O)** The high-magnification image of the area indicated by the red arrow in M. **(P)** Leaf rolling index (LRI) of WT and *dh2-1* leaves. **(Q, R)** Leaf length and leaf width of WT and *dh2-1* leaves. **(S)** Number of leaf veins of WT and *dh2-1* leaves. **(T, U)** total bulliform cell (bc) number **(T)** and area **(U)** between vascular bundle of WT and *dh2-1* leaves. flag, second upper and third upper leaves at the grain-filling period were used. For statistical data, error bars indicate standard deviation (SD). <0.0001 < ***p < 0.001,****p < 0.0001 by two-tailed t-test. ltm, large tumor-like mastoids; stm, small tumor-like mastoids; bc, bulliform cells. The red arrow represents the bulliform cells. Bars = 10 cm in A; 5 mm in **(B, C, I, J)**; 50 μm in **(D, E, K, L)**; 200 μm in F and M; 100 μm in **(G, H, N, O)**.

Under normal physiological conditions, the WT leaf blade was flat (**Figures 3A–C**), whereas the *dh2-1* mutants showed narrow and abaxially rolled leaves throughout the whole growth period (**Figures 3A, I, J**). At the mature period, the LRIs of the top three leaves were investigated between the WT and *dh2-1* mutants. The results showed that the LRIs of the top three leaves of *dh2-1* mutants were significantly increased compared with that of the WT (**Figure 3P**). Meanwhile, it was observed that the width of all top three leaves of *dh2-1* mutants were significantly decreased compared with that of the WT. We also counted the number of small and large vascular bundles in the flag leaves of the WT and *dh2-1* mutants. Compared with the the WT, the number of small and large vascular bundles in the flag leaves of the *dh2-1* mutants was significantly reduced (**Supplementary Figure 3S**). However, it was also found that the length of all top three leaves of *dh2-1* mutants showed no obvious difference compared with that of the WT (**Figures 3Q, R**).

For further clarifying the defects of *dh2-1* leaves at the histological level, the SEM and freezing microtome sections were conducted. Firstly, by using SEM, there were no obvious differences in both adaxial and abaxial surfaces between the WT and *dh2-1* mutant leaves (**Figures 3D, E, K, L**). Next, by analyzing the cross-sections, it was found that both the number and area of bulliform cells on the adaxial surface in *dh2-1* mutants were increased significantly, between large and small veins, and among small veins, compared with those of the WT (**Figures 3F, G, H, M–O, T, U**). As a result, the leaves in *dh2-1* were rolled abaxially. We also observed that the numbers of both large and small veins in *dh2-1* were significantly decreased compared with that of the WT, accounting for the narrow leaves. In general, in *dh2-1* leaves, the adaxial cell feature was disordered and cell differentiation along the medial–lateral direction was repressed, which means that *DH2* also played an important role in the establishment of leaf polarity in rice.

Besides exhibiting abaxially rolled leaves and rod-like lemmas, *dh2-1* showed a series of growth and development defects. Compared with WT, the plant height of *dh2-1* was slightly reduced (**Supplementary Figure S3A**), the panicle length was significantly reduced (**Supplementary Figures S3B, G**), and the number of primary and secondary branches, the number of spikelets per panicle, the number of grains per panicle, and the seed setting rate were decreased (**Supplementary Figures S3H–L**). Meanwhile, we noted that mature grains of *dh2-1* were also affected. Both the length and width of grain and brown grain of *dh2-1* were significantly reduced, which resulted in the decrease of the 1,000-grain weight of *dh2-1* (**Supplementary Figures S3C–E, F, M–R**). Taken together, the abnormal development of lateral organs has a significant effect on rice yield.

### 3.4 Similarity of defects in the *dh2-2* mutants to those in the *dh2-1* mutants

The *dh2-2* mutants showed defects in morphology, histology, and agronomy that were very similar to those of the *dh2-1* mutants (**Supplementary Figures S1**–**S6**). However, some differences were observed. *dh2-2* spikelets were also classified into two types. In type I spikelets, the degree of narrowing of the lemma in *dh2-2* was stronger than that of *dh2-1*, and *dh2-2* produced an elongated rod-like awn at the top of lemma (**Supplementary Figures S1B1**; **Figure 1B1**). In the same planting environment, the changes in *dh2-2* mutants are more severe compared to those of *dh2-1* mutants. The spikelet of *dh2-2* mutants usually presented a completely rod-shaped lemma, while the lemma of *dh2-1* mutants was only slightly narrowed (**Supplementary Figure S1C1**; **Figure 1B1**). Approximately 75% of spikelets per panicle of *dh2-2* had a type II lemma, and approximately 25% of spikelets per panicle of *dh2-2* had a type I lemma. As to spikelets of *dh2-1*, mostly still had a type I lemma per panicle, and almost have no type II lemma, while approximately 10% of spikelets were still normal (**Supplementary Figures S5A–D**). The LRIs of the top three leaves of *dh2-2* mutants were higher than those of *dh2-1* mutant leaves (**Supplementary Figure S4P**; **Figure 3P**). At the same time, the mature brown grains of type I *dh2-1* spikelets had a complete grain morphology, while the morphology of the mature brown grains of *dh2-2* mutants had changed. The shape of the brown grains of *dh2-2* mutants was similar to that of water droplets (**Supplementary Figures S6D, E**, **S3D, F**). Taken together, these observations demonstrate that the *dh2-2* mutant showed more severe defects than the *dh2-1* mutant.

### 3.5 *DH2* encodes a rice argonaute 7 protein

**Figure 4:**
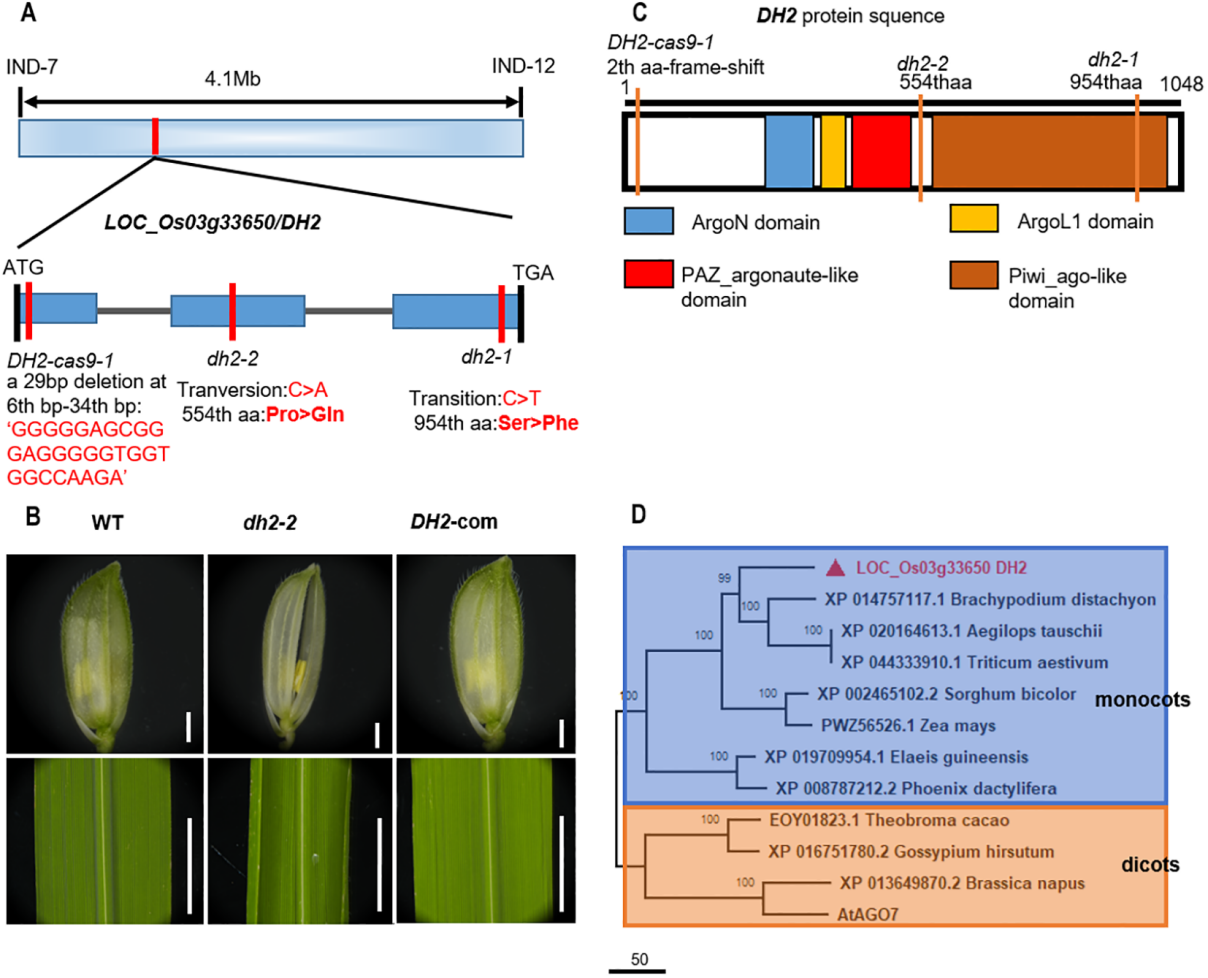
Genetic and complementary analysis of *DH2*. **(A)** Schematic illustration of the genomic structure of *DH2*. **(B)** Genetic complementation of *DH2*. **(C)** Diagram of the conserved domain of *DH2*. **(D)** Phylogenetic tree analysis. The phylogenetic tree was performed using the neighbor-joining method, and bootstrap support values calculated from 1,000 replicates are given at the branch nodes. Bars = 500 mm in B.

Previous studies showed that *DH2* was located between the INDEL markers of IND7 and IND12 on chromosome 3, and there are a total of 13 annotation genes related to flower development within this interval (Guo et al., 2013). In the present study, we performed gene cloning and sequence alignment analysis among those 13 annotation genes between WT and *dh2* mutants. Sequencing analysis showed that a C–T transition in the 2,861st base of the third exon of *LOC_Os03g33650* occurred in *dh2-1*, which causes the replacement of the amino acid Ser to Phe. Meanwhile, in *dh2-2*, there was a C-to-A transversion at the 1,661st base of the second exon of *LOC_Os03g33650*, leading to a Pro–Gln substitution of the 554th amino acid (**Figure 4A**).

To further determine whether the phenotype of *dh2* was due to the mutation of *LOC_Os03g33650*, we constructed the complementary vector containing the 7,623-bp genomic sequence of WT *LOC_Os03g33650* and introduced it into *dh2-1* mutants. It was found that in positive transgenic lines, the mutation phenotype of *dh2-1* was restored completely. Therefore, the results verified that the *DH2* gene was *LOC_Os03g33650* (**Figure 4B**).

To further investigate the function of *DH2*, we conducted phylogenetic tree analysis on *DH2* in Arabidopsis and rice. The results show that *DH2* encodes a rice Argonaute-like 7 protein (**Supplementary Figure S7A**), which has four conserved domains, Piwi_ago-like domain, PAZ_argonaute_like domain, ArgoL1 domain, and ArgoN domain (**Figure 4C**). The Piwi_ago-like domain is the C-terminal portion of Argonaute7, which provides the 5′ anchoring of the guide RNA and the catalytic site for slicing. PAZ_argonaute_like domain functions as a nucleic acid binding domain, with a strong preference for single-stranded nucleic acids (RNA or DNA) or RNA duplexes with single-stranded 3′ overhangs. Phylogenetic tree analysis showed that *DH2* and its homologous genes were widely present, and highly conserved in monocotyledonous and dicotyledonous plants. Those homologous genes could be classified into two subgroups: monocots and dicots. Therefore, it indicated that *DH2* encodes an OsAGO7 protein and may play a similar role to *AtAGO7* (**Figure 4D**).

### 3.6 Overexpression of *DH2* causes adaxially rolled leaves

**Figure 5:**
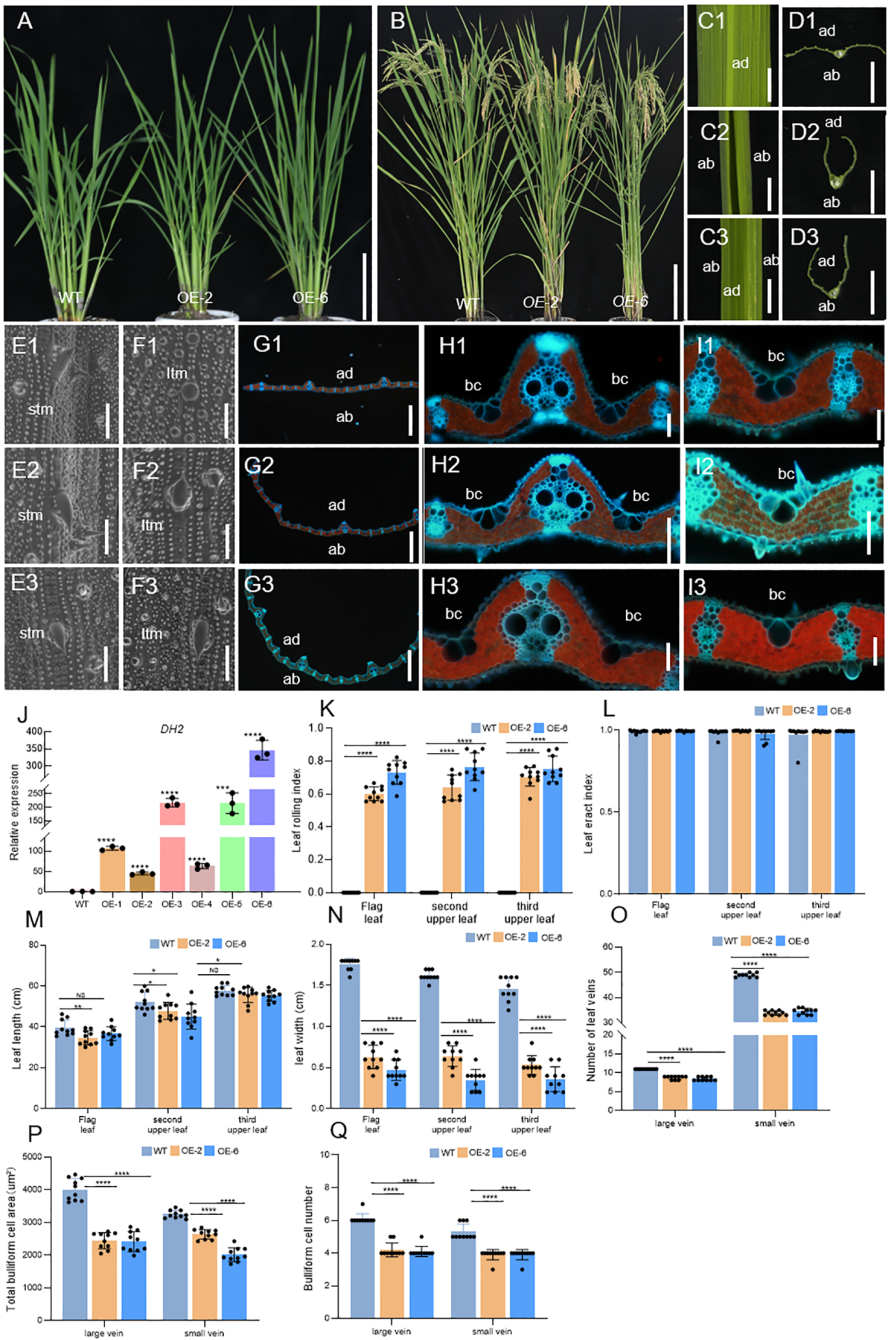
Comparison of WT and OE-DH2 lines (OE-2,6) in terms of plant morphology in seeding stage **(A)**, plant morphology in grain-filling stage **(B)**. **(C, D)** Free-hand cross-sections of WT **(C1, D1)** and OE-2 **(C2, D2)**, OE-6 **(C3, D3)** flag leaf. **(E, F)** SEM observations of the adaxial **(E)** and abaxial **(F)** surface of leaves in WT **(E1, F1)** and OE-2 **(E2, F2)**, and OE-6 **(E3, F3)** in the seeding stage. **(G–I)** Transverse sections of the leaf blade of WT **(G1, H1, I1)** and OE-2 **(G2, H2, I2)** and OE-6 **(G3, H3, I3)** at the seedling stage. **(J)** Relative expression of *DH2* in WT and OE lines leaves in the tillering stage. **(K, L)** Leaf rolling index (LRI) and leaf erect index (LEI) of WT and OE-DH2 (OE-2, OE-4, and OE-6) leaves. **(M, N)** Leaf length and leaf width of WT and OE-DH2 (OE-2, OE-4, and OE-6) leaves. **(O)** Number of leaf veins of WT and OE-DH2 (OE-2, OE-4, and OE-6) leaves. **(P, Q)** Total bulliform cell (bc) area **(P)** and number **(Q)** between vascular bundle of WT and OE-DH2 (OE-2, OE-4, and OE-6) leaves. Flag, second upper and third upper leaves at the grain-filling period were used. For statistical data, error bars indicate standard deviation (SD). 0.01< *p < 0.05, 0.001 < **p < 0.01, <0.0001 < ***p < 0.001, ****p < 0.0001 by two-tailed *t*-test. NS, not significant. Bar = 10 cm in **(A, B, D)**; 100 μm in **E–J**. ltm, large tumor-like mastoids; stm, small tumor-like mastoids; bc, bulliform cells.

Upon analyzing the phenotype of complementary transgenic plants, it was interesting that some of these lines showed adaxially curled leaves, which was just the opposite of abaxially curled leaves in loss-of-function mutants of *DH2*, in addition to being able to restore the phenotype of the *dh2-1* mutant (**Figures 5A–D**). When conducting genetic complementation experiments, we used pCAMBIA1300 vector containing the 2x35s promoter, which significantly increases the expression of the *DH2*. We further detected the expression level of *DH2* in these complementary transgenic lines, OE1 to OE6, and WT. The results showed that in OE1, OE3, OE5, and OE6 lines, the expression of *DH2* increased by at least 100 times, while OE2 and OE4 increased approximately 50 times (**Figure 5J**). Then, it was suggested that adaxially curled leaves in these transgenic plants might be caused by the overexpression of *DH2*.

To further clarify the function of *DH2*, we analyzed the phenotypes of overexpression lines OE-2 and OE-6 in detail. During the mature period, the LRIs of the top three leaves of OE lines were significantly increased. In OE-2, OE-4, and OE-6 lines, the average values of the LRIs of the top three leaves are 50%, 70%, and 75%, respectively (**Figure 5K**), but the LEIs of the top three leaves of OE lines have no obvious changes (**Figure 5L**). In addition, the width and number of large or small veins of leaves in OE lines were extremely significantly decreased while the length of leaves was slightly decreased or unchanged, compared with those in the WT (**Figures 5M–O**). Next, by using SEM, it was found that the adaxial surface of OE-2 and OE-6 was similar to that of WT. However, in the abaxial surface of OE-2 and OE-6, prickly hair cells were massively and regularly produced on the surface of dumbbell cells, while it was rare in the abaxial surface of WT but generally occurred in the adaxial surface of WT (**Figures 5E1–E3, F1–F3**). Finally, in the freezing microtome sections of the leaves in WT, OE-2, and OE-6, it was observed that The leaf of OE-2 and OE-6 curled adaxially, and the number and area of bulliform cells on the adaxial surface between large and small veins, and among small veins, decreased significantly compared with that in WT (**Figures 5G1-G3, H1–H3, I1–I3, P, Q**).

Therefore, the results mentioned above showed that overexpression of *DH2* could cause defects on the development of abaxial–adaxial and medial–lateral axes, further verifying that *DH2* played an important role in the establishment of leaf polarity in rice.

### 3.7 Overexpression of *DH2* affects panicle and grain development

Moderate leaf rolling, which is helpful to establish ideal plant architecture, plays a key role in contributing to the high yields in rice. To evaluate the leaf rolling of overexpression of *DH2* in breeding, we investigated the agronomy traits of the WT and overexpression lines.

**Figure 6:**
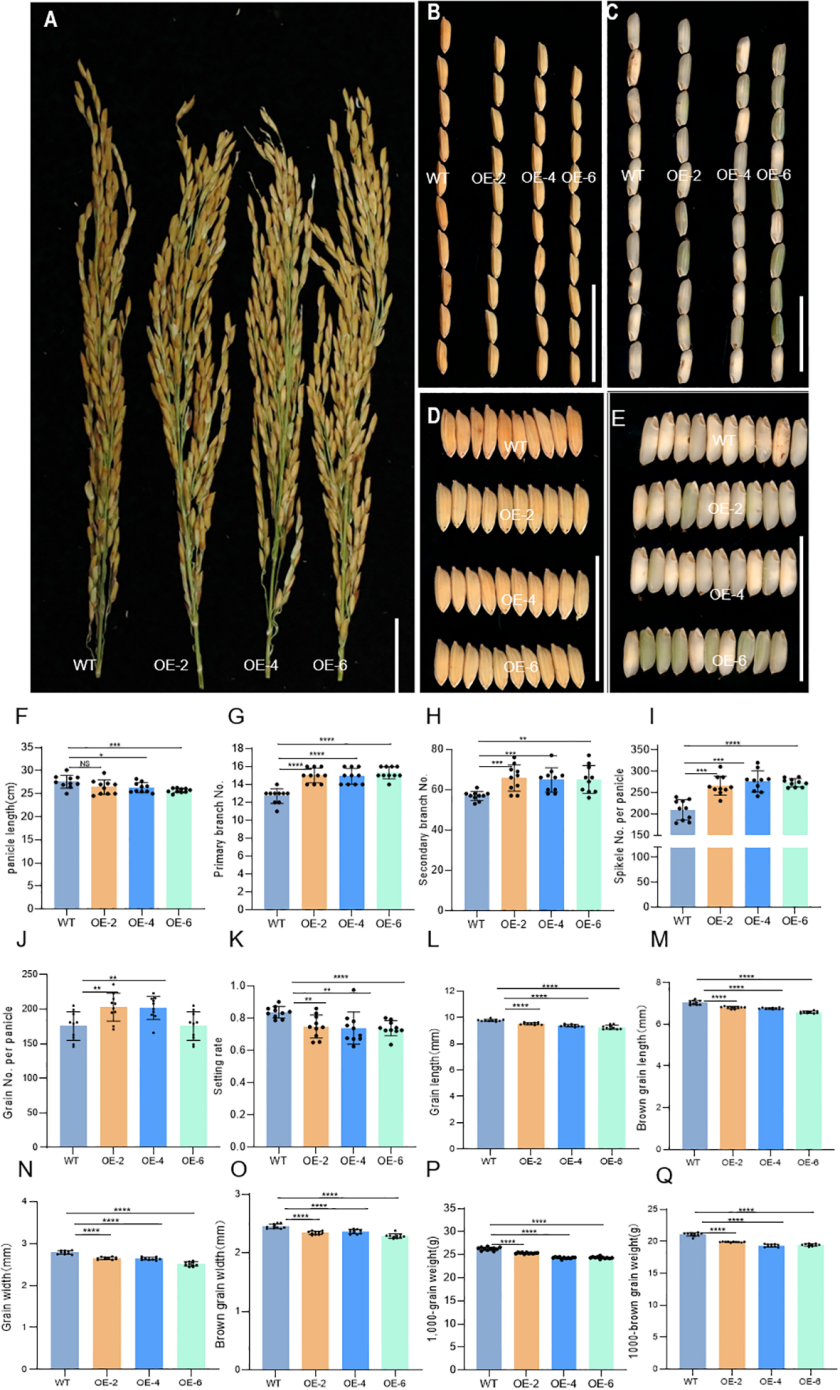
Phenotypic characterization of the wild-type J10 (WT) and *DH2* overexpression (OE-*DH2*) lines (OE-2, OE-4, and OE-6). **(A)** Panicle branch architectures of WT and *OE-DH2* lines. **(B)** Comparison of grain morphology length between WT and *OE-DH2* lines. **(C)** Comparison of brown rice morphology length between WT and *OE-DH2* lines. **(D)** Comparison of grain morphology width between WT and *OE-DH2* lines. **(E)** Comparison of brown rice morphology width between WT and *OE-DH2* lines. **(F)** Panicle length. **(G)** Number of primary branch. **(H)** Number of secondary branch. **(I)** Spikelet number per panicle. **(J)** Grain number per panicle. **(K)** Setting rate. **(L)** Grain length. **(M)** Brown grain length. **(N)** Grain width. **(O)** Brown grain width. **(P)** 1,000-grain weight. **(Q)** 1,000-brown grain weight. For statistical data, error bars indicate standard deviation (SD). 0.01 < *p < 0.05, 0.001< **p < 0.01, <0.0001 < ***p <0.001, ****p < 0.0001 by two-tailed *t*-test. NS, not significant. Bar = 5 cm in **(A–E)**.

It was found that in OE lines, panicle morphology and grain development were changed. Compared with the WT, the panicle length of OE-2, OE-4, and OE-6 significantly decreased (**Figures 6A, F**). However, the number of primary branches, secondary branches, spikelets per panicle, and grain per panicle of OE-2, OE-4, and OE-6 increased significantly. The setting rates of OE-2, OE-4, and OE-6 were gently decreased (**Figures 6G–K**). The length and width of grain and brown grain of OE-2, OE-4, and OE-6 decreased, while the weight of 1,000 grains and brown grains was decreased in the OE lines (**Figures 6B–E, L–Q**). The results showed that overexpression of *DH2* may affect the development of rice panicles and grain.

### 3.8 *DH2* affects the expressions of *tasiR-ARF* and *OsARFs* on the lemma

In order to further understand the functions of *DH2* gene, we examined the spatial expression patterns of *DH2* by RT-qPCR. The results indicated that *DH2* was expressed mainly in leaf blades and young panicle, while it showed a very low expression level in root, shoot, and leaf sheaths. Furthermore, in spikelets, it was found that *DH2* expressed mainly in lemma but not in palea, lodicule, and pistil (**Supplementary Figures S8A, B**). Then, the expression pattern was consistent with the role of *DH2* in regulating the development of leaf blades and lemma.

In Arabidopsis, *AtAGO7*, the *DH2* homolog, has been verified to play a role in the production and silencing complex formation of *tasiR-ARFs* and then mediate the post-transcriptional gene silencing of *AtARF3/4* by specially degrading the mRNA of *ARF3/4* on the adaxial side of leaves, thus contributing to the establishment of adaxial identity (Allen et al., 2005; Axtell et al., 2006; Montgomery et al., 2008; Peragine et al., 2004; Iwakawa et al., 2021; Sakurai et al., 2021).

**Figure 7:**
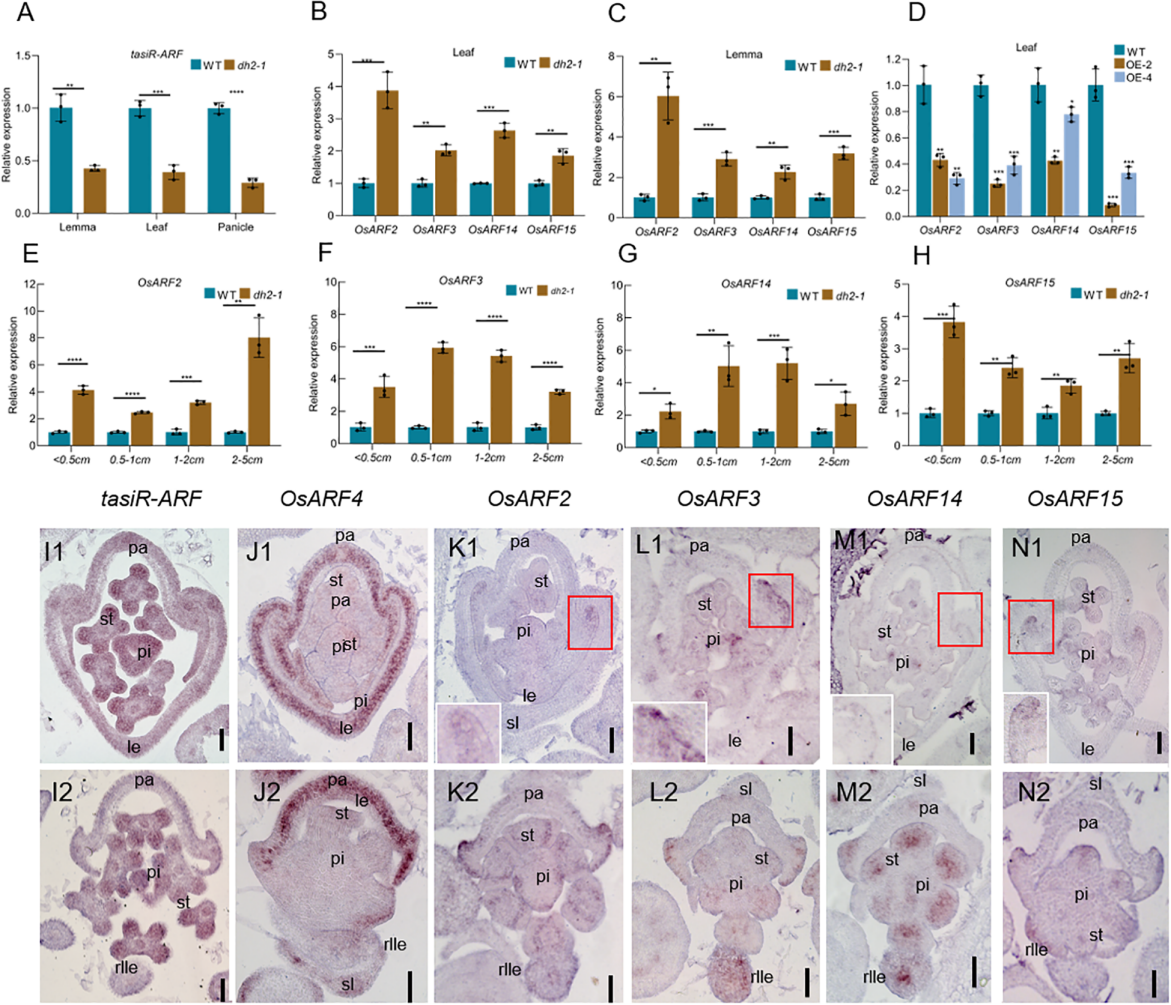
Expression of *OsARFs* and *tasiR-ARFs* in WT and *dh2-1*. **(A–C)** The relative expression of *OsARF2*, *OsARF3*, *OsARF14*, and *OsARF15* in the leaf and lemma of WT, *dh2-1*, OE-2, and OE-4 using RT-qPCR. **(C–G)** The relative expression of *OsARF2*, *OsARF3*, *OsARF14*, and *OsARF15* in the different panicle lengths of WT and *dh2-1* using RT-qPCR. **(H)** The relative expression of *tasiR-ARFs* in the leaf, lemma and panicle. **(I1–N1, I2–N2)** *In situ* hybridization analysis of *tasiR-ARFs, OsARF4, OsARF2*, *OsARF3*, *OsARF14*, and *OsARF15* in the spikelet of WT and *dh2-1*, **(I1-N1)**, WT; (I2–K2) *dh2-1*. *ACTIN (LOC_Os03g50885)* and U6 were used as an internal control. *OsARF4* was used as a positive control. Data are mean ± SD (*n* = 3 biological replicates). sl. sterile lemma; le, lemma; pa, palea; st, stamen; pi, pistil; rlle, rod-like lemma. 0.01 < *p < 0.05, 0.001< **p < 0.01, <0.0001 < ***p <0.001, ****p < 0.0001 by two-tailed *t*-test. Bars = 50 μm in **(I1–N1, I2–N2)**. The image in the upper right corner is an enlarged view of the red square area in **(K1, L1, M1, N1)**.

In this study, the expression of *tasiR-ARFs* was detected firstly by RT-qPCR. The results showed that it was decreased significantly in the leaf, lemma, and panicle of *dh2-1* compared with that in WT (**Figure 7A**). Then, we detected the expression of *OsARF2*, *OsARF3*, *OsARF14*, and *OsARF15*, the homologs of the *AtARF3/4*, between *dh2-1* mutants and the WT (**Supplementary Figure S9A**). The results showed that their expression was significantly increased in the leaf and lemma in *dh2-1* compared with that in the WT (**Figures 7B, C**), whereas their expression in the leaf of OE-2 and OE-4 was significantly decreased compared with that in the WT (**Figure 7D**). Meanwhile, expression levels of these genes in young panicles were detected. The results showed that their expression was significantly increased in panicles with different lengths, compared with those in the WT (**Figures 7E–H**). Therefore, these data indicated that *DH2/OsAGO7* also mediated the formation of *tasiR-ARFs* and then repressed the expression of *ARFs* in rice, which may be quite conserved in monocots and dicots.

To further clarify the expression pattern of *tasiR-ARFs* and its targets *OsARFs*, we conducted *in situ* hybridization. The results showed that *tasiR-ARFs* are widely expressed in the floral organ primordia of the WT (**Figure 7I1**). However, there was no obvious signal of *tasiR-ARFs* in the rod-like lemma primordia in *dh2-1* mutants, while it was still expressed in the other floral organ primordia in *dh2-1* mutants (**Figure 7I2**). In the WT spikelets, *OsARF2, OsARF3, OsARF14*, and *OsARF15* shared a similar expression pattern, mainly expressing on the abaxial surface of the margin region of palea, and weak signals of these genes could be detected in the stamens and pistils, whereas they were not almost expressed in the lemma primordia (**Figures 7K1, L1, M1, N1**). In *dh2-1* spikelets, the signal of *OsARF2, OsARF3, OsARF14*, or *OsARF15* was slightly enhanced in the abaxial surface of the margin region of palea, pistil, and stamen. Most importantly, strong ectopic signals of these genes were detected in the rod-shaped lemma primordia (**Figures 7K2, L2, M2, N2**). In addition, we also detect the expression of *OsARF4.* In the WT flowers, *OsARF4* specifically expresses in the lemma and palea primordia. However, it could not be detected in the rod-like lemma primordia in the *dh2-1* spikelets. We used *OsARF4* as a positive control to provide strong support for the reliability of *in situ* hybridization experiments (**Figures 7J1, J2**). Together, these results indicated that *DH2* was mainly responsible for repressing the expression of *OsARF2, OsARF3, OsARF14*, and *OsARF15* in lemma as a way of mediating the production of *ta-siRNA*.

## 4 Discussion

### 4.1 *DH2*-mediated *tasiR-ARFs* synthesis and *ARFs* repression affect the polarity of lateral organs in rice

*tasiR-ARFs* are unique to plants and play an important role in their growth and development, which includes the regulation of the adaxial–abaxial polarity of lateral organs, as well as SAM formation (Nagasaki et al., 2007; Toriba et al., 2010; Arikit et al., 2013; Garcia et al., 2006; Fahlgren et al., 2006; Adenot et al., 2006). AGO7 specifically loads miR390 to target *TAS3* transcripts, leading to their cleavage, which primarily determines the synthesis of mature *tasiR-ARFs* although several proteins like SGS3, RDR6, and DCL4 are also important (Allen et al., 2005; Axtell et al., 2006; Montgomery et al., 2008; Peragine et al., 2004; Iwakawa et al., 2021; Sakurai et al., 2021). In Arabidopsis and rice, the mutations of *AGO7, RDR6*, and *DCL4* lead to the decreased accumulation of *tasiR-ARFs* and increased expression of target gene *ARFs*, and affect the adaxial–abaxial polarity of leaf and/or floral organs (Xu et al., 2006; Li et al., 2005; Fahlgren et al., 2006; Yoon et al., 2010; Marin et al., 2010; Nagasaki et al., 2007; Toriba et al., 2010; Song et al., 2012).

In this study, *DH2* encodes *OsAGO7* in rice. In *dh2* mutants, the rod-shaped lemma with abaxial epidermis was observed, meaning the adaxial side of lemma was abaxialized. It was also found that the leaf showed outward rolling in *dh2* mutants, due to the development defects of bulliform cell at the adaxial side. Moreover, the expression of *tasiR-ARFs* was reduced in the lemma, leaf, and spikelet primordia, and the expression of *OsARF2, OsARF3, OsARF14, and OsARF15* was significantly increased. Therefore, it seemed that *AGO7–tasiR-ARFs–ARFs* was conserved in the establishment of the polarity of lateral organs between rice and Arabidopsis.

However, we found that the transcripts of four *OsARF* genes showed organ speciality but not organ polarity in lemma in the present study. More narrowly, they were all expressed in palea but not in lemma in the WT, whereas they were all ectopically expressed in rod-shaped lemma. Although the four genes show an organ polarity expression pattern (expressing at the abaxial side of margin region of palea) in the WT, they showed the same expression pattern in the *dh2* palea. As a result, we also observed no obvious defects in the *dh2* palea. Meanwhile, we also found that *tasiR-ARFs* were expressed in the whole lemma and palea but not in the abaxial side. This means that there was no opposite expression of *tasiR-ARFs* and *ARFs* in adaxial–abaxial of lemma. In previous studies about the pathway of *AGO7–tasiR-ARFs–ARFs*, they commonly used qRT-PCR and/or Northern blot to detect the expression of *tasiR-ARFs* and *ARFs* in lateral organs; thus, it was difficult to clarify how their expression patterns were between the WT and mutants (Zhang et al., 2023; Itoh et al., 2000; Nagasaki et al., 2007; Liu et al., 2007; Song et al., 2012). According to our data, we believe that the pathway of *OsAGO7–tasiR-ARFs* in rice was more likely involved in the development of the entire lemma, and not only the abaxial side, by restricting ectopic expression of *OsARFs* in the whole lemma, which was different with that in lateral organs of Arabidopsis. In *dh2* mutants, while *tasiR-ARFs* was decreased, *OsARF2, OsARF3*, and *OsARF15* were ectopically expressed in the whole lemma, while *OsARF14* was ectopically expressed at the center of the rod-like lemma, which finally resulted in the rod-shaped abaxialized lemma.

### 4.2 *DH2* affects the rice yield

Moderate leaf rolling, which is helpful to establish ideal plant architecture, plays a key role contributing to high yields in rice. As the organ of photosynthesis, leaf morphology is directly related to factors such as photosynthesis area and effective photosynthetic efficiency (Xu et al., 2018; Du et al., 2018). The development of hull (lemma and palea) affects grain shape, because it limits the length and width of grain (Xiang et al., 2015; Yu et al., 2018). Therefore, reasonable leaf and hull morphologies are important factors for high yields in rice.

In this study, most agronomy traits in *dh2* mutants, like height, number of spikelets per panicle, setting rate, and grain weight, were all decreased compared with WT, indicating that the *dh2* mutation negatively affected yield, due to changes in the morphology of leaf and lemma. Interestingly, we identified some overexpression of *DH2* lines in the complementary test. These lines showed adaxially rolling leaves throughout the whole growth period, which was opposed to abaxially rolling leaves in the *dh2* mutants. In a previous study, an R05 mutant showed the upregulated expression of the *OsAGO7* gene, caused by the T-DNA inserting into the promoter region of the gene. The R05 mutant showed an upward rolling leaf and an increasing LRI, which enhanced erect-leaf habits and elevated the value of LEI (Shi et al., 2007). Now, it is common that the appropriate rolled leaf is regarded as a critical element for the ideal rice phenotype (Yuan, 1997; Chen et al., 2001; Zhang et al., 2009). Moderate leaf rolling helps to maintain the erectness of leaves, which can increase the light transmission rate and light saturation point without affecting the light compensation point, resulting in a well-proportioned leaf area for photosynthesis (Duncan, 1971; Zhang et al., 2009). Moderate leaf rolling improves photosynthetic efficiency, accelerates dry-matter accumulation, increases yield, reduces the solar radiation on leaves, and decreases leaf transpiration under drought stress (Lang et al., 2004; Zhang et al., 2009).

In our study, it was found that the number of spikelet/grain per panicle was significantly increased in the overexpression of *DH2* lines, while the setting rates of overexpression of *DH2* lines decreased slightly. The length and width of grain and brown grain of overexpression of *DH2* lines decreased, while the weight of 1,000 grains and brown grains decreased in the overexpression of *DH2* lines. In future breeding, utilizing the overexpression of *DH2* to increase the number of spikelets per panicle while improving grain size and seed setting rate to increase yields by other genes is a promising approach. Thus, it is of great practical value to enhance *DH2* expression in future molecular design breeding.

## 5 Conclusion

We identified two alleles, named *degenerated hull 2-1* and *degenerated hull 2-2* (*dh2-1* and *dh2-2*), which encode a relatively conservative AGO7 protein in plants, which is involved in the synthesis of *tasiR-ARFs*. The production of *tasiR-ARF* depends on the function of *AGO7*, and *tasiR-ARFs* form a silencing complex with *AGO7* to inhibit downstream *ARFs*, thereby regulating the lateral organ polarity development. In rice, the decrease of *tasiR-ARFs* in *dh2-1* and *dh2-2*; the increase of *OsARF2, OsARF3, OsARF14*, and *OsARF15*; and the disruption of their polarity distribution of lateral organ indicate that *DH2*-mediated *tasiR-ARFs* synthesis regulates lateral organ polarity development in rice.

## Data Availability

Sequence data from this article can be accessed in the GenBank database under the following accession numbers: DH2 (OsAGO7), ACTIN (LOC_Os03g50885), and accession numbers for OsARF2, OsARF3, OsARF14, and OsARF15 are AB071291, AK072330, NM_001062573, and AK062170, respectively. Locus identifications in the Rice Genome Annotation Project Database are as follows: DH2/OsAGO7 (LOC_Os03g33650), OsARF2 (LOC_Os01g48060), OsARF3 (LOC_Os01g54990), OsARF14 (LOC_Os05g43920), and OsARF15 (LOC_Os05g48870).

## Supplementary material

Supplementary Figure 1 Phenotypes of spikelet in the wild-type (WT) and degenerated hull 2-2 (dh2-2). A1, the complete spikelet of the WT. A2, the lemma was removed. the lemma and palea was removed in A3. A4, the complete spikelet of the WT. A5 and A6, surface characters of palea (A5) and lemma (A6) of WT spikelet. A7, cross section of WT. A8 and A9, transverse sections of palea (A8) and lemma (A9) of WT spikelet. B1, the complete spikelet of the dh2-2 with a typeI lemma. B2, the lemma was removed. the lemma and palea was removed in B3. B4, the complete spikelet of the dh2-2 with a typeI lemma. B5 and B6, surface characters of palea (A5) and lemma (A6) of the dh2-2 with a typeI 2’lemma. B7, cross section of dh2-2 with a typeI lemma. A8 and A9, transverse sections of palea (A8) and lemma (A9) of dh2-2 with a typeI lemma. C1, the complete spikelet of the dh2-2 with a type II lemma. C2, the lemma was removed. the lemma and palea was removed in C3. C4, the complete spikelet of the dh2-2 with a type II lemma. C5 and C6, surface characters of palea (C5) and lemmaa (C6) of the dh2-2 with a type II lemma. C7, cross section of dh2-2 with a type II lemma. C8 and C9, transverse sections of palea (C8) and lemma (C9) of dh2-2 with a type II lemma. le, lemma; rlle, rod-like lemma; pa, palea; sl, sterile lemma; lo, lodicule; st, stamen; pi, pistil; mrp, marginal region of palea; bop,body of palea. Bars=1000 μm in A1-A3, A5, A6, B1-B3, B5, B6, C1-C3, C5, C6. Bars=5 mm in A4, B4, C4. Bars=200um in A7, B7, C7. Bars=50um in A8, A9, B8, B9, C8, C9.

Supplementary Figure 2 Scanning electron micrographs of florets at early developmental stages in the wild-type (WT) and degenerated hull 2-2 (dh2-2). **(A–D)**, WT. **(E–F)**, spikelet of dh2-2 with a typeI lemma. **(I–L)**, spikelet of dh2-2 with a type II lemma. **(A, E, I)**, Sp4; **(B, F, J)**, Sp5-Sp6; **(C, G, K)**, Sp7; **(D, H, L)**, Sp8. rg. rudimentary glume; sl, sterile lemma; le, lemma; rlle, rod-like lemma; pa, palea; st, stamen; pi, pistil; fm, flower meristem. Bars=500 μm in **(A–L)**.

Supplementary Figure 3 Agronomic traits WT and dh2-1 mutant. **(A, B)** comparison of WT **(A)** and dh2-1 **(B)** in terms of plant morphology. C, comparison of panicle of WT and dh2-1. **(C, D)** length and width comparison of grain morphology of WT and dh2-1. **(E, F)** length and width comparison of brown grain morphology of WT and dh2-1. Plant length **(G)**. Primary branch number per panicle **(H)**. Secondary branch number per panicle **(I)**. Spikelet number per panicle **(J)**. Grain number per panicle **(K)**. Setting rate **(L)**. Grain length **(M)**. Brown grain length **(N)**. Grain width **(O)**. Brown grain width **(P)**. 1000-grain weight **(Q)**. 1000-brown grain weight **(R)**. For statistical data, error bars indicate standard deviation (SD). 0.01<*P<0.05, 0.001< **P<0.01, <0.0001<***P <0.001, ****P<0.0001 by two-tailed t test. Bar=10cm in **(A, B)**; 5cm in **(C)**; 1cm in **(D, E)**.

Supplementary Figure 4 Phenotypes of leaves of WT and dh2-2 mutant. **(A)** phenotypes of the WT and dh2-2 at the jointing stage. **(B)**, flag leaf of WT. **(C)**, free-hand cross sections of WT flag leaf. **(D and E)**, SEM observations of the adaxial **(D)** and abaxial **(E)** surface of leaves in WT. **(F)**, transverse sections of the leaf blade of WT at the seedling stage. **(G,H)**, the high-magnification image of the area in the black box in **(F, I)**, flag leaf of dh2-2. **(J)**, free-hand cross sections of dh2-2 flag leaf. K and L, SEM observations of adaxial **(K)** and abaxial **(L)** surface of leaves in dh2-2. **(M)**, transverse sections of the leaf blade of dh2-2 at the seedling stage. **(N, O)**, the high-magnification image of the area in the black box in **(M)**. **(P)** Leaf rolling index (LRI) of WT and dh2-2 leaves. **(Q, R)**, Leaf length and leaf width of WT and dh2-2 leaves. **(S)**, Number of leaf veins of WT and dh2-2 leaves. **(T, U)**, total bulliform cell (bc) number **(T)** and area **(U)** between vascular bundle of WT and dh2-2 leaves. Flag, second upper and third upper leaves at the grain-filling period were used. For statistical data, error bars indicate standard deviation (SD). 0.01<*P<0.05, 0.001< **P<0.01, <0.0001<***P <0.001, ****P<0.0001 by two-tailed t test. ltm, large tumor-like mastoids; stm, small tumor-like mastoids; blc, block-like cells; bc, builform cells; bcl, builform cell-like. The red arrow represents the builform cells. Bars=10cm in **(A)**; 0.5cm in **(B, C, I, J)**; 50um in **(D, E, K, L)**; 200um in **(F, M)**; 100um in **(G, H, N, O)**.

Supplementary Figure 5 dh2-1 and dh2-2 exhibited abnormal spikelets. **(A)** comparison of panicle of J10 and dh2-1. **(B)** comparison of panicle of 1B and dh2-2. **(C)** proportion of abnormal spikelet of dh2-1. C, proportion of abnormal spikelet of dh2-2.

Supplementary Figure 6 Agronomic traits WT and dh2-2 mutant. A, B, comparison of WT **(A)** and dh2-2 **(B)** in terms of plant morphology. **(C)**, comparison of panicle and internode of WT and dh2-2. **(D, E)**, length and width comparison of brown grain morphology of WT and dh2-2. Plant height **(F)**. Tillering number **(G)**. Panicle length **(H)**. Internode length **(I)**. Primary branch number per panicle **(J)**. Secondary branch number per panicle **(K)**. Spikelet number per panicle **(L)**. Grain number per panicle **(M)**. Setting rate **(N)**. 1000-grain weight **(O)**. For statistical data, error bars indicate standard deviation (SD). 0.01<*P<0.05,0.001< **P<0.01, <0.0001<***P <0.001,****P<0.0001 by two-tailed t test. Bar=10cm in **(A, B)**; 5cm in **(C)**; 1cm in **(D, E)**.

Supplementary Figure 7 Phylogenetic tree analysis of AGO protein between rice and Arabidopsis. The phylogenetic tree was performed using the neighbor-joining method, and bootstrap support values calculated from 1000 replicates are given at the branch nodes.

Supplementary Figure 8 Spatiotemporal expression pattern of the DH2. A and B, RT-qPCR of DH2. ACTIN (LOC_Os03g50885) was used as an internal control. spikelet, vegetative organs, and floral organs of WT were used. Data are Mean ± SD (n=3).

Supplementary Figure 9 Phylogenetic tree analysis of ARF protein between rice and Arabidopsis. The phylogenetic tree was performed using the neighbor-joining method, and bootstrap support values calculated from 1000 replicates are given at the branch nodes.

Supplementary Table 1 Primers in this study.

Supplementary Table 2 Information of plant proteins used for phylogenetic analysis.
