# Supplementary figures and images for: DH2-dependent trans-acting siRNAs regulate leaf and lemma development in rice
Source: Front Plant Sci. 2025 Jan 27;15:1534038. doi: 10.3389/fpls.2024.1534038 (PMC11808002; doi:10.3389/fpls.2024.1534038)

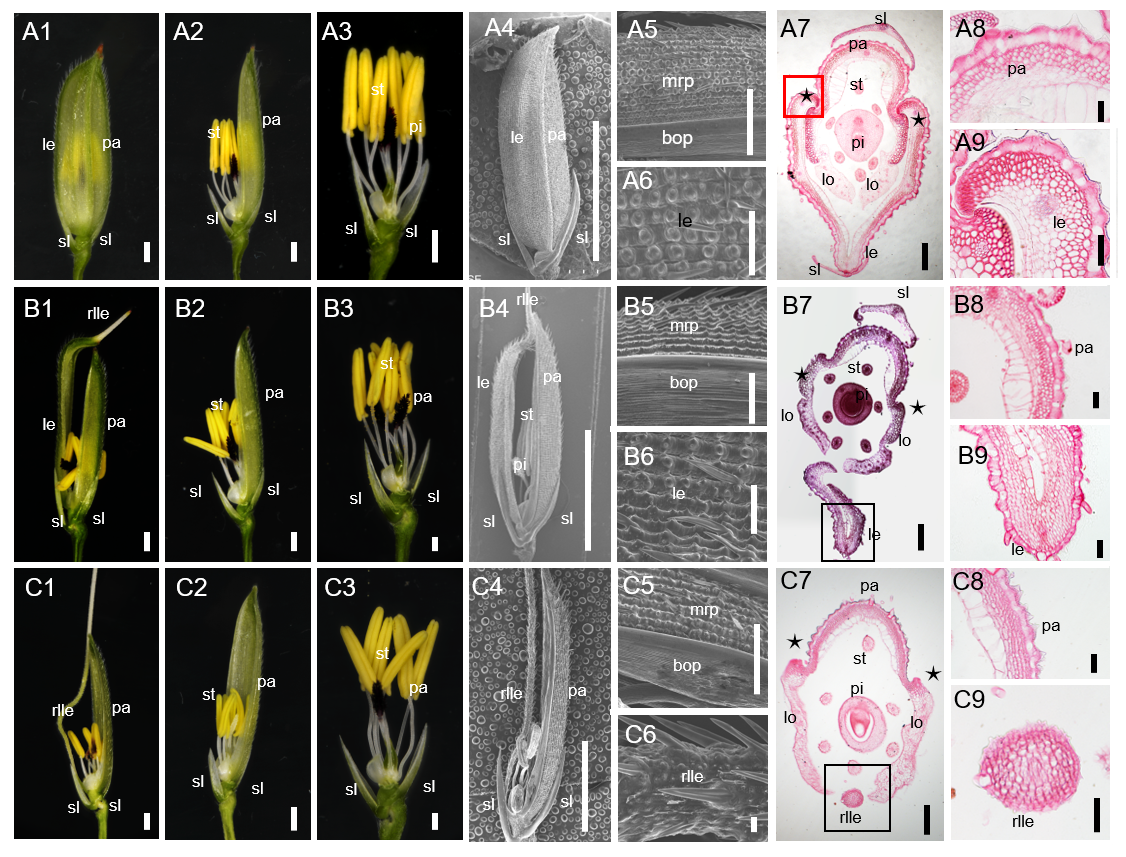

Supplement: Supplementary Figure 1 — Phenotypes of spikelet in the wild-type (WT) and degenerated hull 2-2 (dh2-2). A1, the complete spikelet of the WT. A2, the lemma was removed. the lemma and palea was removed in A3. A4, the complete spikelet of the WT. A5 and A6, surface characters of palea (A5) and lemma (A6) of WT spikelet. A7, cross section of WT. A8 and A9, transverse sections of palea (A8) and lemma (A9) of WT spikelet. B1, the complete spikelet of the dh2-2 with a typeI lemma. B2, the lemma was removed. the lemma and palea was removed in B3. B4, the complete spikelet of the dh2-2 with a typeI lemma. B5 and B6, surface characters of palea (A5) and lemma (A6) of the dh2-2 with a typeI 2’lemma. B7, cross section of dh2-2 with a typeI lemma. A8 and A9, transverse sections of palea (A8) and lemma (A9) of dh2-2 with a typeI lemma. C1, the complete spikelet of the dh2-2 with a type II lemma. C2, the lemma was removed. the lemma and palea was removed in C3. C4, the complete spikelet of the dh2-2 with a type II lemma. C5 and C6, surface characters of palea (C5) and lemmaa (C6) of the dh2-2 with a type II lemma. C7, cross section of dh2-2 with a type II lemma. C8 and C9, transverse sections of palea (C8) and lemma (C9) of dh2-2 with a type II lemma. le, lemma; rlle, rod-like lemma; pa, palea; sl, sterile lemma; lo, lodicule; st, stamen; pi, pistil; mrp, marginal region of palea; bop,body of palea. Bars=1000 μm in A1-A3, A5, A6, B1-B3, B5, B6, C1-C3, C5, C6. Bars=5 mm in A4, B4, C4. Bars=200um in A7, B7, C7. Bars=50um in A8, A9, B8, B9, C8, C9. [file SupplementaryFigure1.tif]

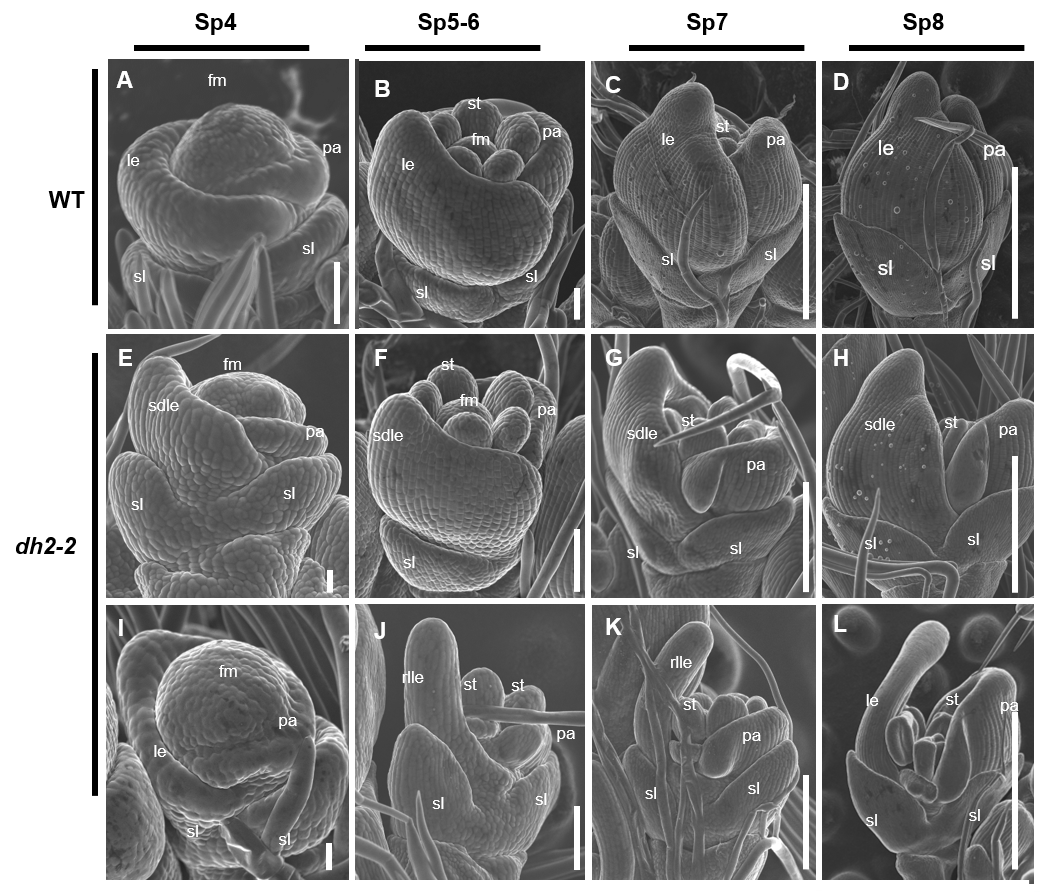

Supplement: Supplementary Figure 2 — Scanning electron micrographs of florets at early developmental stages in the wild-type (WT) and degenerated hull 2-2 (dh2-2). (A–D), WT. (E–F), spikelet of dh2-2 with a typeI lemma. (I–L), spikelet of dh2-2 with a type II lemma. (A, E, I), Sp4; (B, F, J), Sp5-Sp6; (C, G, K), Sp7; (D, H, L), Sp8. rg. rudimentary glume; sl, sterile lemma; le, lemma; rlle, rod-like lemma; pa, palea; st, stamen; pi, pistil; fm, flower meristem. Bars=500 μm in (A–L). [file SupplementaryFigure2.tif]

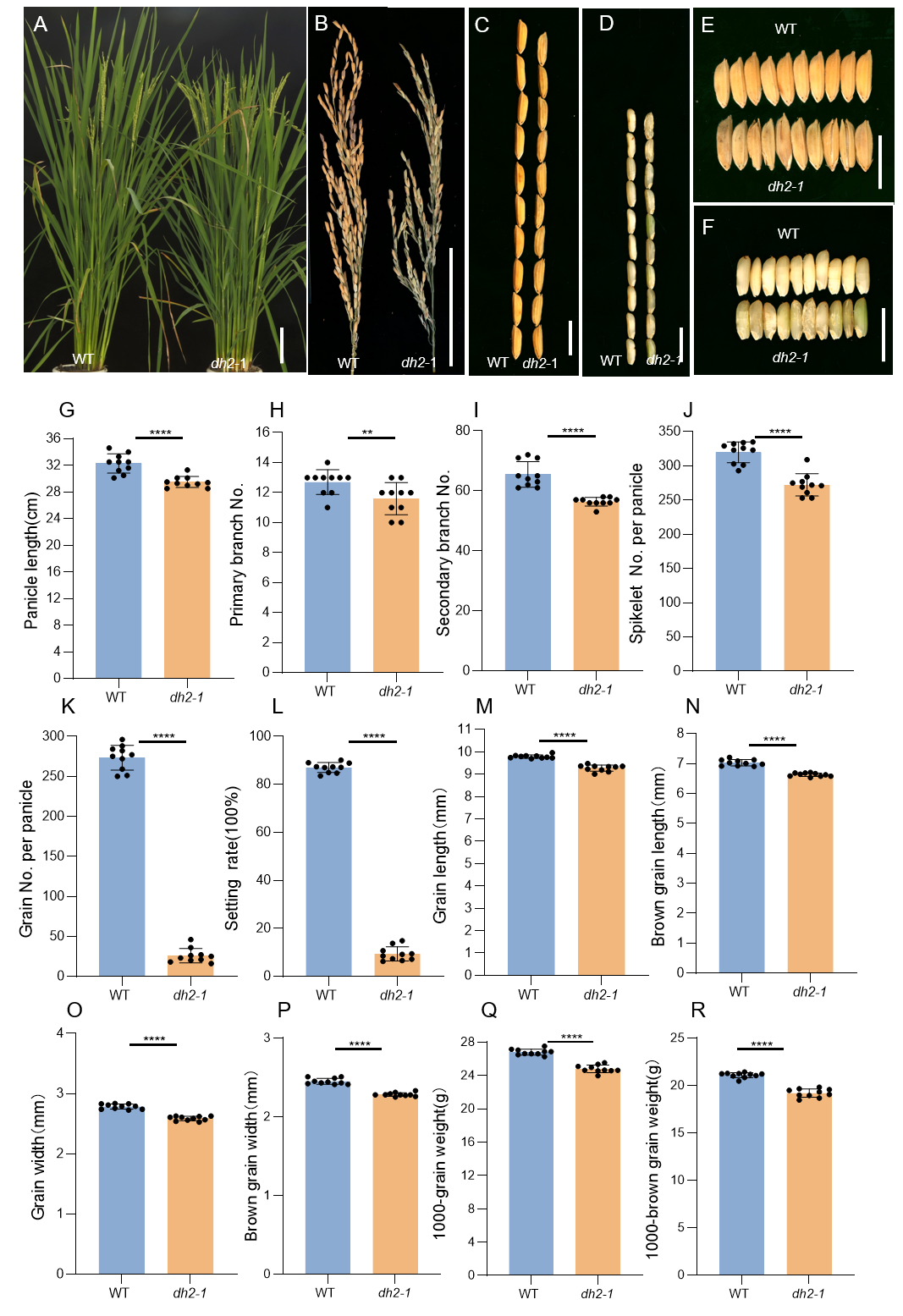

Supplement: Supplementary Figure 3 — Agronomic traits WT and dh2-1 mutant. (A, B) comparison of WT (A) and dh2-1 (B) in terms of plant morphology. C, comparison of panicle of WT and dh2-1. (C, D) length and width comparison of grain morphology of WT and dh2-1. (E, F) length and width comparison of brown grain morphology of WT and dh2-1. Plant length (G). Primary branch number per panicle (H). Secondary branch number per panicle (I). Spikelet number per panicle (J). Grain number per panicle (K). Setting rate (L). Grain length (M). Brown grain length (N). Grain width (O). Brown grain width (P). 1000-grain weight (Q). 1000-brown grain weight (R). For statistical data, error bars indicate standard deviation (SD). 0.01<*P<0.05, 0.001< **P<0.01, <0.0001<***P <0.001, ****P<0.0001 by two-tailed t test. Bar=10cm in (A, B); 5cm in (C); 1cm in (D, E). [file Image3.tif]

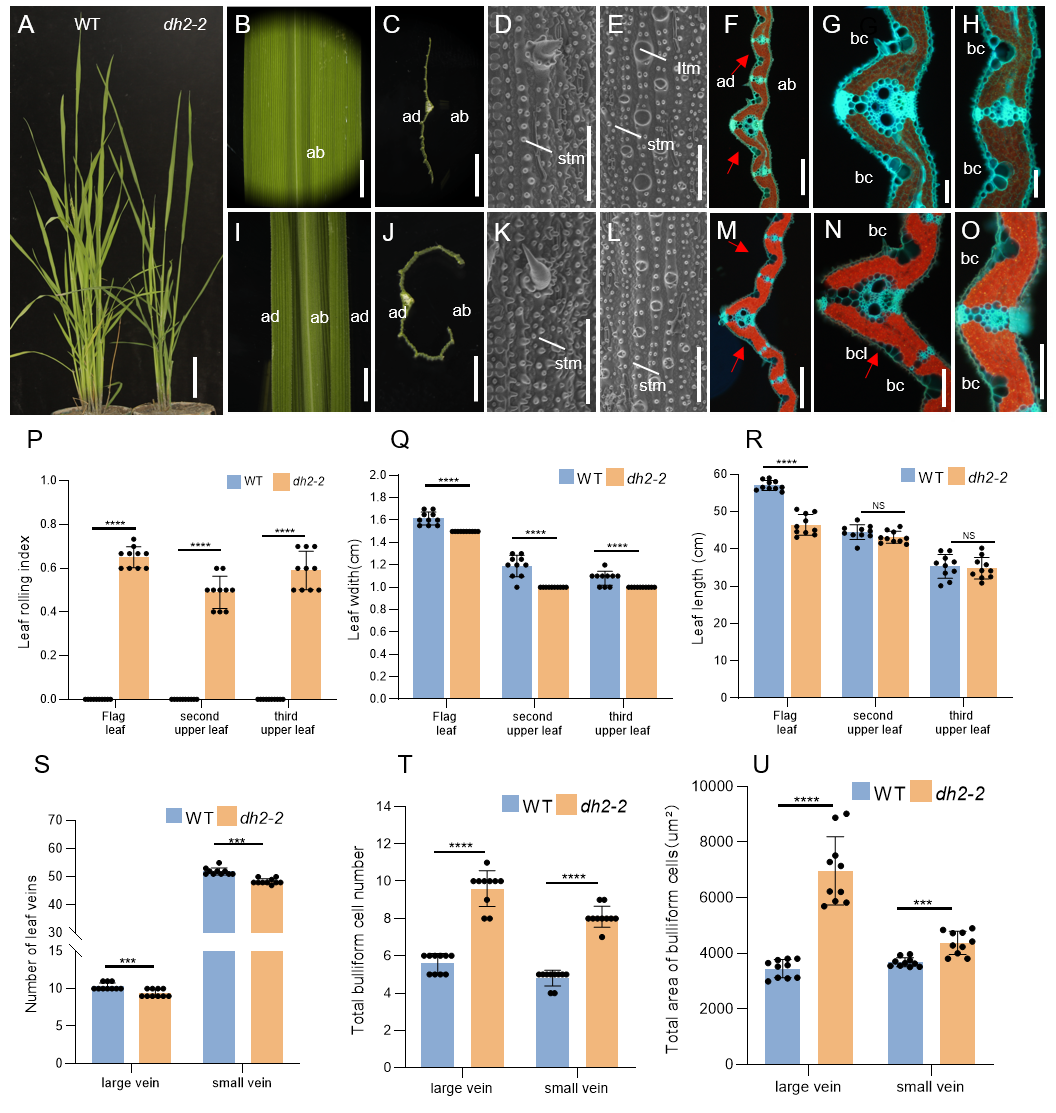

Supplement: Supplementary Figure 4 — Phenotypes of leaves of WT and dh2-2 mutant. (A) phenotypes of the WT and dh2-2 at the jointing stage. (B), flag leaf of WT. (C), free-hand cross sections of WT flag leaf. (D and E), SEM observations of the adaxial (D) and abaxial (E) surface of leaves in WT. (F), transverse sections of the leaf blade of WT at the seedling stage. (G,H), the high-magnification image of the area in the black box in (F, I), flag leaf of dh2-2. (J), free-hand cross sections of dh2-2 flag leaf. K and L, SEM observations of adaxial (K) and abaxial (L) surface of leaves in dh2-2. (M), transverse sections of the leaf blade of dh2-2 at the seedling stage. (N, O), the high-magnification image of the area in the black box in (M). (P) Leaf rolling index (LRI) of WT and dh2-2 leaves. (Q, R), Leaf length and leaf width of WT and dh2-2 leaves. (S), Number of leaf veins of WT and dh2-2 leaves. (T, U), total bulliform cell (bc) number (T) and area (U) between vascular bundle of WT and dh2-2 leaves. Flag, second upper and third upper leaves at the grain-filling period were used. For statistical data, error bars indicate standard deviation (SD). 0.01<*P<0.05, 0.001< **P<0.01, <0.0001<***P <0.001, ****P<0.0001 by two-tailed t test. ltm, large tumor-like mastoids; stm, small tumor-like mastoids; blc, block-like cells; bc, builform cells; bcl, builform cell-like. The red arrow represents the builform cells. Bars=10cm in (A); 0.5cm in (B, C, I, J); 50um in (D, E, K, L); 200um in (F, M); 100um in (G, H, N, O). [file Image4.tif]

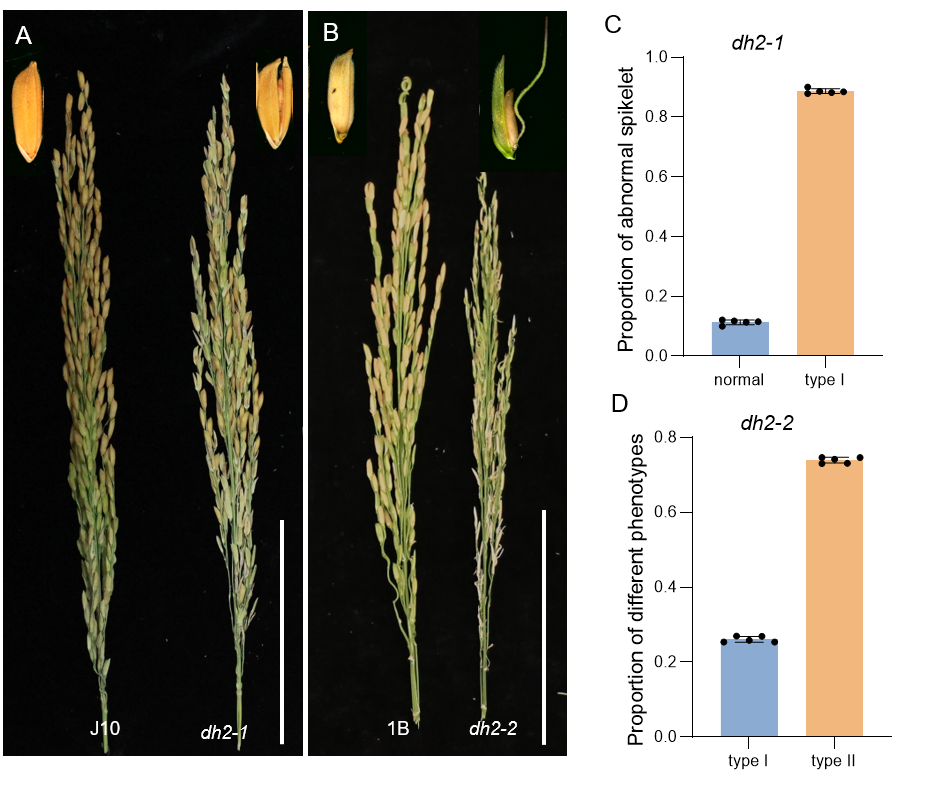

Supplement: Supplementary Figure 5 — dh2-1 and dh2-2 exhibited abnormal spikelets. (A) comparison of panicle of J10 and dh2-1. (B) comparison of panicle of 1B and dh2-2. (C) proportion of abnormal spikelet of dh2-1. C, proportion of abnormal spikelet of dh2-2. [file Image5.tif]

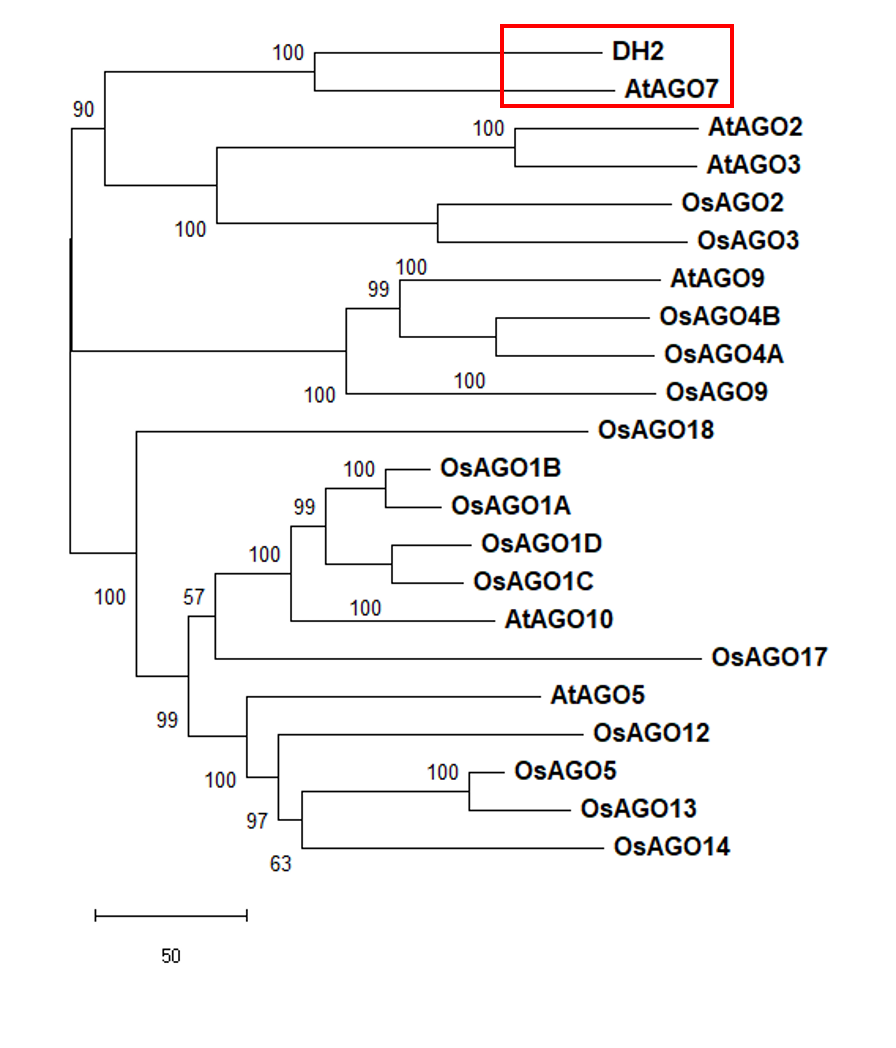

Supplement: Supplementary Figure 7 — Phylogenetic tree analysis of AGO protein between rice and Arabidopsis. The phylogenetic tree was performed using the neighbor-joining method, and bootstrap support values calculated from 1000 replicates are given at the branch nodes. [file Image7.tif]

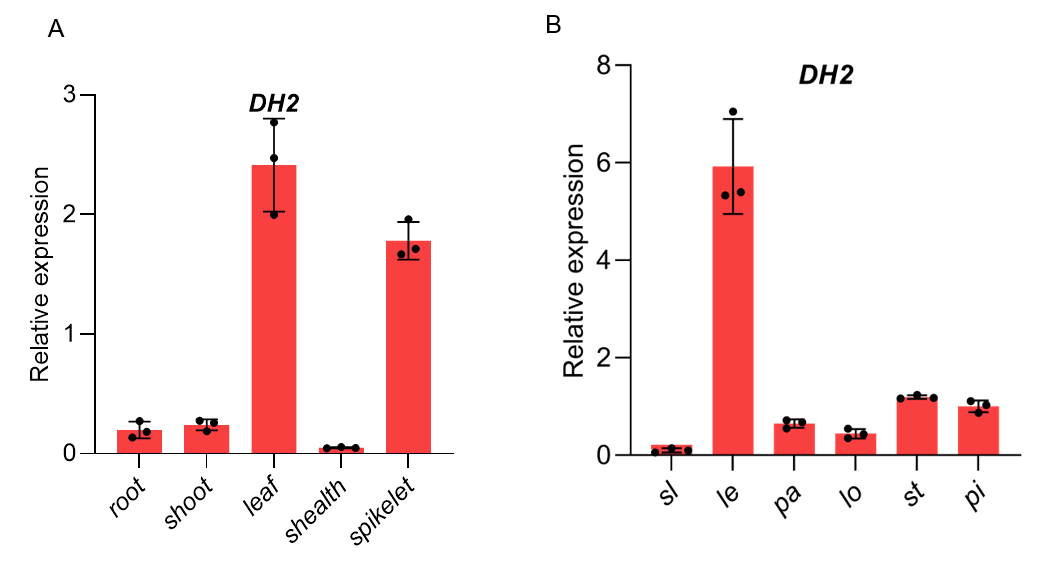

Supplement: Supplementary Figure 8 — Spatiotemporal expression pattern of the DH2. A and B, RT-qPCR of DH2. ACTIN (LOC_Os03g50885) was used as an internal control. spikelet, vegetative organs, and floral organs of WT were used. Data are Mean ± SD (n=3). [file Image8.tif]

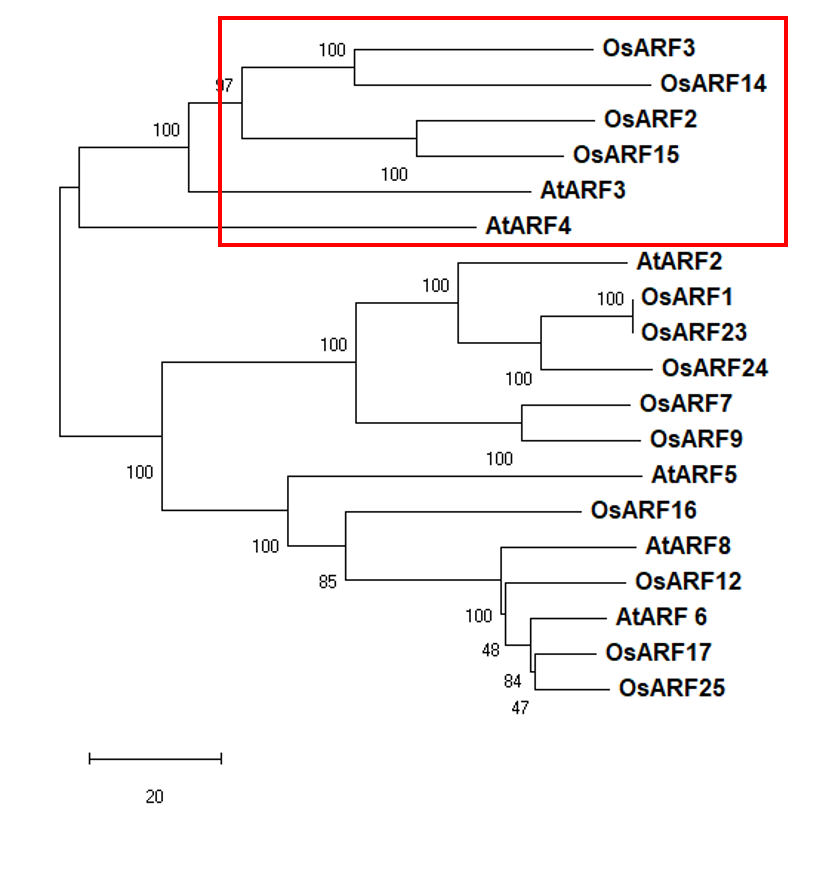

Supplement: Supplementary Figure 9 — Phylogenetic tree analysis of ARF protein between rice and Arabidopsis. The phylogenetic tree was performed using the neighbor-joining method, and bootstrap support values calculated from 1000 replicates are given at the branch nodes. [file Image9.tif]
